# Incorporating household structure and demography into models of endemic disease

**DOI:** 10.1098/rsif.2019.0317

**Published:** 2019-08-07

**Authors:** Joe Hilton, Matt J. Keeling

**Affiliations:** 1 MathSys CDT, Mathematics Institute, University of Warwick, Coventry CV4 7AL, UK; 2 Zeeman Institute (SBIDER), University of Warwick, Coventry CV4 7AL, UK; 3 Mathematics Institute, University of Warwick, Coventry CV4 7AL, UK; 4 School of Life Sciences, University of Warwick, Coventry CV4 7AL, UK

**Keywords:** childhood diseases, mathematical modelling, endemic diseases, demography, family structure

## Abstract

The spread of infectious diseases is intimately linked with the strength and type of contact between individuals. Multiple observational and modelling studies have highlighted the importance of two forms of social mixing: age structure, where the likelihood of interaction between two individuals is determined by their ages; and household structure, which recognizes the much stronger contacts and hence transmission potential within the family setting. Age structure has been ubiquitous in predictive models of both endemic and epidemic infections, in part due to the ease of assessing someone’s age. By contrast, although household structure is potentially the dominant heterogeneity, it has received less attention, in part due to an absence of the necessary methodology. Here, we develop the modelling framework necessary to predict the behaviour of endemic infections (which necessitates capturing demographic processes) in populations that possess both household and age structure. We compare two childhood infections, with measles-like and mumps-like parameters, and two populations with UK-like and Kenya-like characteristics, which allows us to disentangle the impact of epidemiology and demography. For this high-dimensional model, we predict complex nonlinear dynamics, where the dynamics of within-household outbreaks are tempered by historical waves of infection and the immunity of older individuals.

## 1. Introduction

Epidemiological modelling plays a vital role in public-health planning, both in terms of generic understanding of infectious disease transmission and control, as well as in terms of detailed predictions for particular situations. The foundation of these models is the compartmental epidemic model of Kermack & McKendrick [1], but modern predictive models generally seek to capture additional heterogeneities within the population [2,3]. These heterogeneities often reflect differential risk structure in terms of the transmission dynamics; high impact examples include age-stratified risk structure [4,5] and spatial structure [6,7]. Producing robust and accurate predictions often relies on an informed choice of which forms of structure to include and the reliable inference of associated parameters.

Age-structured models have a rich history, particularly for endemic childhood infections (such as measles) where they have been used to capture the greater rates of transmission between school-age children and the impact of opening and closing of schools [4,5]. In this context, and before vaccination, age structure is a major risk factor as only relatively young children are likely to be susceptible to the disease, with older age cohorts having already been infected and hence having developed immunity. Age-structured models have also been vital in epidemic settings (such as influenza pandemics) where they have been an integral part of disease forecasting [8,9]. For both endemic and epidemic infections, age-structured models benefit from the ease with which the age of cases is recorded and the number of recent studies which quantify social contact patterns with reference to age stratification [10,11]. These studies provide a growing body of empirical data with which to parametrize age-structured models—overcoming the limitations of inference that is based sole on age-distribution data [5]. In addition, it has been suggested that Bayesian hierarchical models can be used to estimate contact patterns from country-level socioeconomic indicators in the absence of detailed survey data [12], vastly increasing the number of settings amenable to this form of age-structured predictive disease modelling.

Models that recognize household transmission are dominated by the more theoretical literature, linking household structure to the final distribution of infection following a single outbreak [6,13]. In such models, households are treated as discrete units characterized by their internal composition with respect to the infection (i.e. two susceptibles, one infected and one recovered) and it is generally assumed that transmission is strong within the household but weak and homogeneous between households. This model formulation has provided an important understanding of optimal control in such structured populations [6,13–15], while related approaches have commonly been used to infer the strength of within-household mixing [16,17]. More complex approaches incorporate further levels of structure to model school- and workplace-based contacts [18–20], and have been used to study the efficacy of interventions such as school closure [21]. We motivate the use of household models for endemic infections by the following question: is a child in a large household more at risk of infection than one in a small household? Clearly having an older sibling (compared to being an only child) increases the risk of infection, as older children are a conduit of infection into the home. However, older siblings could already have been infected, and so will be immune and cocoon the younger child. Understanding this complex interaction between infection and household demography requires the use of mathematical models.

To date, only a limited number of simulation-based approaches have been able to successfully combine age and household structure [8,9] or to model the impact of household structure for endemic infections [22,23]. In this paper, we formulate a *household infectious disease model with demography*: a continuous-time deterministic model of infectious disease which incorporates both age- and household structure into its transmission dynamics and captures the evolution of households over time as events in a Markov process.

## 2. Methods

Here, we outline the fundamental processes under-pinning our model, we focus on describing the mechanics of the model while the detailed mathematical description is in the electronic supplementary material.

The household infectious disease model with demography combines a Markov chain model for the slow evolution of a household with a Markovian SIR disease model that captures internal transmission within the household, homogeneous between-household transmission and age-structured transmission. The state of a single household is defined by the quadruple (*S*, *I*, *R*, *k*), where *S*, *I* and *R* are the number of susceptible, infectious and recovered individuals in the household, and *k* is an integer-valued counter which determines the demographic evolution of the household. At specific values of *k*, demographic events occur which cause individuals to be added to or removed from the household; *k* is generally incremented (at exponentially distributed time intervals) but can also be reset to allow repeated demographic events. The demographic status of the household is entirely determined by *N*( = *S* + *I* + *R*) and *k*, and these two values can be encoded as a single integer *T*.

To avoid confusion with the states of our Markov chain, and by analogy with the concept of an age class, we refer to the demographic configuration encoded by *T*(*N*, *k*) as the household’s *demographic class*. Fixing a maximum household size *N*_max_ defines a finite range of values of *T* and thus a finite state space for the combined demographic–infectious process. We assume an asymptotically large population of households, such that the proportion of households ($\underline{H}$) in each state obeys a set of deterministic ordinary differential equations (ODEs); this allows the calculation of population-level epidemiological quantities from the household-level state distribution. Specifically, the population-level disease prevalence $\bar{I}$ of the disease is equal to the expected number of infectious individuals per household divided by the expected household size:

$$\bar{I}=\frac{\sum_{S,I,R,k}IH_{S,I,R,k}}{\sum_{S,I,R,k}(S+I+R)H_{S,I,R,k}},$$

and the infectious prevalence stratified by demographic class *T*(*N*, *k*), denoted by ${\bar{I}}_{T}$, is given by the expected number of infectious cases in a household conditioned on that household being in demographic class *T*, divided by *N*:

$${\bar{I}}_{T}=\frac{\sum_{S+I+R=N,k}IH_{S,I,R,k}}{\sum_{S+I+R=N,k}NH_{S,I,R,k}},$$

where *H* refers to the proportion of households in a given state. The proportion of households in demographic class *T*, denoted by *H*_*T*_, is calculated by summing over all states (*S*, *I*, *R*, *T*):

$$H_{T}=\sum_{S+I+R=N_{T}}H_{S,I,R,T},$$

where *N*_*T*_ is the number of individuals in a household of class *T*. To fully define the model, we also need to calculate the probability that a child has infectious status *R* at the instant they leave home, which we denote by *P*_*R*_.

Following Ross *et al.* [24], we denote the household state distribution of the system at time *t* by $\underline{H}(t)$ and the transition matrix by $Q(\underline{H})$. The dependence of Q on $\underline{H}$ arises whenever external conditions impinge upon the household dynamics, as such the dependence is through the three key population-level variables $\bar{I}$, ${\bar{I}}_{T}$ and *P*_*R*_. $\bar{I}$ and ${\bar{I}}_{T}$ determine the transmission into the household from homogeneous and age-dependent mixing respectively, while new households (counter value *k* = 1) are formed of susceptible and recovered adults drawn from the pool of children leaving home—hence the immune status of these new households is governed by *P*_*R*_. The transition matrix can be decomposed into three components, $Q(\underline{H})=Q_{\mathrm{Demo}}(\underline{H})+Q_{\mathrm{Int}}+Q_{\mathrm{Ext}}(\underline{H})$, each of which is described in more detail below. $Q_{\mathrm{Demo}}$ contains all the rates for demographic events and depends nonlinearly on *P*_*R*_, $Q_{\mathrm{Int}}$ contains all the rates for recovery and internal transmission events and is a constant matrix, and $Q_{\mathrm{Ext}}$ contains all the rates for external transmission events and hence depends linearly on both $\bar{I}$ and ${\bar{I}}_{T}$. The evolution of the state distribution is then determined by the nonlinear set of ODEs

2.1 $$\frac{\text{d}\underline{H}}{\text{d}t}=\underline{H}\;Q(\underline{H}),$$

with the nonlinearity arising from the dependence of $Q_{\mathrm{Demo}}$ and $Q_{\mathrm{Ext}}$ on $\underline{H}$. In disease-free situations, the dynamics simplify to a linear problem

2.2 $$\frac{\text{d}\underline{H}}{\text{d}t}=\underline{H}\;Q_{\mathrm{Demo}}(P_{R}=0),$$

whose long-term equilibrium is given by the eigenvector associated with the largest (zero) eigenvalue of $Q_{\mathrm{Demo}}$.

### 2.1. Demographic model

Our demographic model describes the evolution of a simple nuclear household, beginning at counter value *k* = 1. At *k* = *k*_*B*_, a child is born, adding an extra susceptible individual to the household. The sequence *k* = 1, …, *k*_*B*_ can be repeated a random number of times, with the distribution informed by the family size distribution of the population we wish to model. In this way, multiple children can be born into the household, with *k* only incrementing to *k*_*B*_ + 1 once the last child is born.

The household loses an individual at *k* = *k*_*B*_ + *k*_*L*_, when the eldest child leaves home, and subsequently at *k* = 2*k*_*B*_ + *k*_*L*_, when any younger siblings leave home. The eldest child matures during the interval of *k*_*L*_ steps between when their youngest sibling being born and them leaving home—hence the rate at which the counter *k* moves through these *k*_*L*_ states is dependent on the number of children in the household. The *k*_*B*_ steps between successive children leaving home leads to the same age distribution at leaving for all of the children in a household. The sequence *k* = *k*_*B*_ + *k*_*L*_ + 1, …, 2*k*_*B*_ + *k*_*L*_ is repeated until all of the children have left home; counter values above 2*k*_*B*_ + *k*_*L*_ are associated with elderly couples. When the counter attains its maximum value of *k* = 2*k*_*B*_ + *k*_*L*_ + *k*_*R*_, the household reaches the end of its lifetime and is renewed, with the two remaining individuals in the household being replaced by two new ones whose immunological status is governed by *P*_*R*_ (the proportion of recovered individuals leaving home).

**Figure 1.**
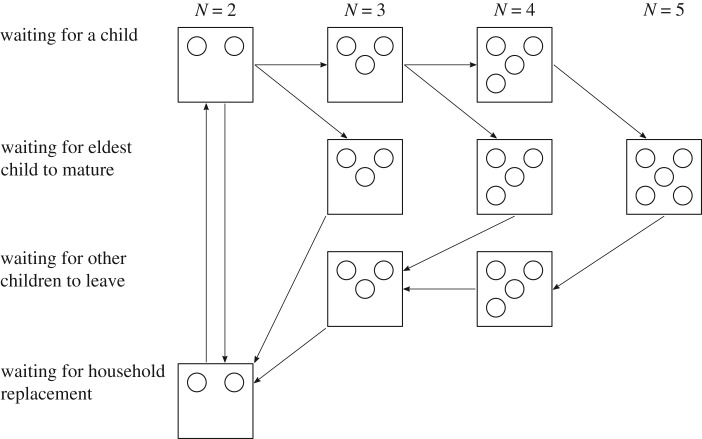
The household demographics is defined by the household size *N* and phase. Each row corresponds to one phase of the household’s lifespan, moving sequentially from the birth phase, to waiting for the oldest child to leave, to waiting for other children to leave, to the reset-and-replacement phase. The intervals between transitions all follow an Erlang distribution. In this example, the maximum household size is 5.

The demographic process is explained in more detail in electronic supplementary material, S4 and is demonstrated schematically in figure 1. The integers *k*_*B*_, *k*_*L*_ and *k*_*R*_ are fixed model parameters and can be interpreted as shape parameters for a set of Erlang distributions which define the waiting periods between demographic events. The rate at which *k* increments depends on the household’s position in the demographic process (i.e. which row of the schematic in figure 1 it is currently on); this allows us to control the mean and the shape parameter of each Erlang waiting time independently.

To fully define the demographic model, we need to specify the infectious status of the individuals who are added to or removed from the household. We will assume for this model that all newborns are susceptible, so there is no maternal immunity or vaccination. The two adults who arrive in the household at *k* = 0 are chosen at random from the pool of children currently leaving home, so that they are independently recovered with probability *P*_*R*_ and susceptible with probability (1 − *P*_*R*_), ignoring the (small) probability that the individuals will be infectious. The calculation of *P*_*R*_ relies on knowing the probability that each parent was recovered at the start of a household’s lifetime, which we call *P*′_*R*_. The ‘current generation’ households will therefore be initiated with an average of 2*P*′_*R*_ recovered individuals, and so if we choose the infectious status of children leaving home by discounting these recovered individuals from consideration:

$$P_{R}=\frac{1}{\sum_{S,I,R}\;H_{S,I,R,T_{\mathrm{leave}}}}\sum_{S,I,R}\;H_{S,I,R,T_{\mathrm{leave}}}\;\frac{R-2P_{R}^{\prime }}{N-2P_{R}^{\prime }},$$

where $H_{S,I,R,T_{\mathrm{leave}}}$ refers to household states where a leave event occurs (*k* = *k*_*B*_ + *k*_*L*_ or *k* = 2*k*_*B*_ + *k*_*L*_). In a population with no previous exposure to infection, *P*′_*R*_ = 0, which admits a simple calculation for *P*_*R*_; when the dynamics are at equilibrium *P*′_*R*_ = *P*_*R*_ allowing us to find the probability recursively using a self-consistency procedure. The results presented in this paper are entirely concerned with either the early growth or equilibrium behaviour of the model, making these two values of *P*′_*R*_ sufficient for our purposes.

Determining the infectious status of fully grown children leaving home follows the same logic as above, but is more complicated as it must be conditioned on the infectious status of the household—for example, it is only possible to removed recovered children if recovered individuals are present in the household. (Full details of the mechanism is given in electronic supplementary material, S4.2).

### 2.2. Epidemiological model

The epidemiological dynamics incorporate three routes of infection, for a household of demographic class *T* and size *N* these are

(1) frequency-dependent internal infection at rate $\beta^{\mathrm{int}}\frac{I}{N-1}S$
(2) homogeneous frequency-dependent external infection at rate $(1-\sigma )\beta^{\mathrm{ext}}\bar{I}S$
(3) age-structured external infection at rate $\sigma \lambda_{T}(\underline{H})S$,

where *β*^int^ and *β*^ext^ are, respectively, the internal and external person-to-person transmission rates, $\bar{I}$ is the population-level prevalence of infection, and $\lambda_{T}(\underline{H})$ is the age-structured force of infection on individuals in a household of demographic class *T* when the state distribution of the population is $\underline{H}$. These three routes of infection result in an identical transition *S* → *I*. Infectious individuals recover and transition *I* → *R* at rate *γ*, leading to exponentially distributed infectious periods. The value of *σ* ∈ [0, 1] defines the relative proportion of unstructured and structured external mixing, tuning between homogeneous external mixing at *σ* = 0 and purely age-structured external mixing at *σ* = 1. Our choice of frequency-dependent internal mixing is motivated by Hope-Simpson’s study on childhood infections, in which the attack rates of measles and mumps are shown to be only weakly dependent on household size [25]. Further support for this choice is offered by the data-driven work of Cauchemez *et al.* [26] which suggests that within-household transmission is substantially closer to the frequency-dependent than to the density-dependent ideal.

The internal transmission rate *β*^int^ is calculated by multiplying the per unit-time transmission rate *τ* by the average time per day spent exposed to within-household contacts, as calculated from contact data. The external transmission rate *β*^ext^ is given by the analogous formula using the average time per day spent exposed to contacts from other households. Age-structured transmission rates for a set of age classes *C*_1_, …, *C*_*K*_ are calculated by multiplying *τ* by the average total duration of contacts between an individual in age class *C*_*i*_ and all individuals in age class *C*_*j*_ who are in different households—we label the resulting transmission rate $\beta_{ij}^{\mathrm{ext}}$. By defining our total external infection rate to be a convex combination of the homogeneous and age-structured infection rates (controlled by the parameter *σ*), we can control the level of structure in our model while keeping the total population-level transmission rate constant. The calculation of *τ* and the required contact durations is covered in electronic supplementary material, S5.

Our approach to age-structured mixing takes the typical who-acquires-infection-from-whom approach first introduced by Schenzle [4] but also requires a mapping between age classes and demographic classes in order to model mixing structured by demographic class. Because the counter *k* increments at exponentially distributed intervals, the time spent in any amalgamation of demographic classes is hypoexponentially distributed. Using this information, we can define a matrix $E=E_{T,i}$ such that *E*_*T*,*i*_ the expected number of individuals in age class *C*_*i*_ in a household of demographic class *T*. As such, the population-level proportion of individuals in age class *C*_*i*_ is then

$$P_{i}=\frac{\sum_{S,I,R,T}H_{S,I,R,T}E_{T,i}}{\sum_{S,I,R,T}H_{S,I,R,T}(S+I+R)}.$$

The force of infection on individuals in a household of type *T* is then determined by summing across all associated age classes multiplied by their interaction with other age classes and the chance that those age classes are infectious:

$$\lambda_{T}=\sum_{i,j}\frac{E_{T,i}}{N_{T}}\beta_{i,j}^{\mathrm{ext}}\sum_{U}H_{U}E_{U,j}\frac{{\bar{I}}_{U}}{P_{\;j}},$$

where the second sum approximates the proportion of infected individuals in age class *C*_*j*_. The full derivation of this equation is given in more detail in electronic supplementary material, S6.

Under suitable parameter choices, our model contains the classic homogeneous mixing model (*β*^int^ = *σ* = 0), an age-structured model (*β*^int^ = 0, *σ* = 1) and a household-structured model as special cases (*σ* = 0). As a set of (high-dimensional) differential equations, the system is numerically tractable, allowing us to study both its early behaviour following invasion and its endemic equilibrium; both of these can be approached with considerable computational efficiency by relying on the matrix structure of the underlying ODEs (equation 1). In particular, we focus on the early growth rate *r* and the household reproduction number R* [6] as measures of early dynamics, as well as the equilibrium of the combined demographic and epidemiological system. We compare the full model to other sub-models (homogeneous, age structured and household structured only) to assess the impact of individual forms of structure and contact heterogeneity; this also informs about the likely problems with predictions from simpler models that ignore particular forms of contact structure.

To clarify the effects of demography and epidemiology, we study two childhood diseases (a measles-like disease and a mumps-like disease which differ in their transmission rate across a contact [25] and infectious period [2] (table 1)) in two different populations (a UK-like population and a Kenya-like population). Our choice of these two populations is motivated by the availability of detailed contact survey data and the pronounced socioeconomic differences between the two countries, which we expect to be reflected in demography and contact behaviour. The POLYMOD study provides contact data for the UK [10] and the study conducted by Kiti *et al.* [11] provides contact data from the region of coastal Kenya covered by the Kilifi Health and Demographic Surveillance System [27]. The demographic transition terms ($Q_{\mathrm{Demo}}$) are parametrized directly from empirical data [28–32], without recourse to any fitting procedure.

**Table 1.** Values of transmission rate, *τ*, and recovery rate, *γ*, for the measles- and mumps-like diseases used throughout the paper. These are calculated from the pairwise secondary attack rate *p* as derived by Hope-Simpson [25] and from the expected infectious periods, *IP* [2]. (All time-scales are in days).

	*p*	*IP*	*τ*	*γ*
measles	0.756	7	1.7822	0.143
mumps	0.311	8	0.4118	0.125

## 3. Results

Throughout, we study the dynamics of infection in the absence of vaccination or other forms of control. We focus on the early invasion dynamics and equilibrium distribution of infection, and how these are impacted by accounting for different transmission heterogeneities.

### 3.1. Demographic equilibrium distribution

**Figure 2.**
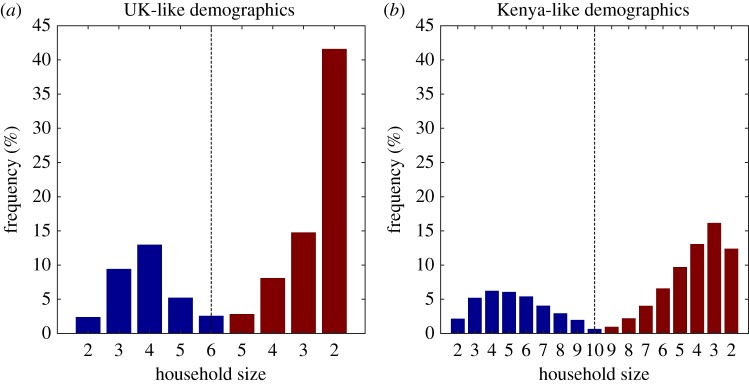
Household size and age distributions for populations with UK-like and Kenya-like parameters. Blue bars correspond to the first two phases of the demographic process outlined in figure 1 (prior to the eldest child leaving home), red bars correspond to the third and fourth phases (after the eldest child has left), so that moving along the *x* axis can be thought of as moving through successive stages in a household’s life.

The household size distribution at demographic equilibrium (the solution of equation (2.2) is illustrated for the UK-like and a Kenya-like populations in figure 2, and is partitioned into younger (blue) and older (red) households. The UK-like population is dominated by older households without children as a result of its relatively long life expectancy and low birth rate, in part a reflection of the high proportion of adults (approx. 17% [29]) not having any children at all. The Kenya-like population features a much higher proportion of households with children, resulting from a combination of a lower life expectancy, a higher average number of children, and comparable inter-birth interval to the UK, which causes adults to spend a much higher proportion of their life living with children compared the UK-like population. These demographic equilibrium solutions form the underlying population structure to which invading and endemic infections are added.

### 3.2. Impact of assumed transmission structure

**Figure 3.**
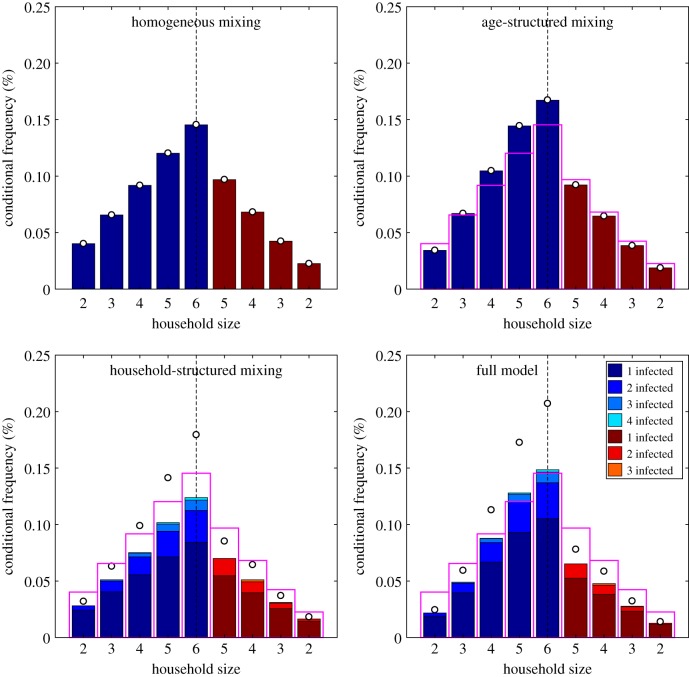
Percentage distribution of cases per household conditional on the size and age of the household, under the four different transmission models. These results are for a mumps-like infection in a UK-like population. For purposes of clarity, we do not plot the percentage of disease-free households since this is several orders of magnitude larger than the other percentages combined. As in figure 2, blue bars correspond to younger households (the first two rows in figure 1) and red to older households (the second two rows in figure 1). The pink open bars correspond to the results from the homogeneous mixing model, which are shown for ease of comparison; the open circles show the total amount of infection in the households accounting for multiple infections.

Initially, we focus on a disease with mumps-like epidemiological parameters, such that the rate of transmission across a contact is relatively low (*τ*_mumps_ = 0.4118 per contact per day), and consider the short- and long-term dynamics in a UK-like population (table 2 and figure 3). We compare the full model (which includes both age-structured and household-structured transmission) to the homogeneous, purely age-structured and purely household-structured sub-models. To fairly compare the four models, the transmission rate *τ* in the three sub-models is re-scaled to achieve the same initial growth rate *r* in all cases—conceptualized as matching the four models to the same early epidemic data and then making predictions about the long-term dynamics.

**Table 2.** Early growth parameters, equilibrium prevalence and childhood infection probability for a mumps-like disease in a UK-like population, for four sub-cases of the household infectious disease model with demography. Mumps-like parameters are chosen for the full age- and household-structured model, with *τ* chosen for the other three cases to give the same early growth rate *r* = 0.121 in all cases. The corresponding household prevalence distributions are visualized in figure 3.

model	*τ*	R*	$\bar{I}\times 10^{4}$	*P*_*R*_
homogeneous mixing	0.3308	2.057	1.756	0.425
age structured	0.3331	2.057	1.788	0.465
household structured	0.4265	2.647	1.748	0.444
full model	0.4118	2.691	1.760	0.480

Parameter values capturing early invasion dynamics into a naive population, and those describing long-term endemicity are listed in table 2. *r* defines the asymptotic rate of early growth, such that Cases ∼ exp(*rt*); while R* (which is the household counterpoint of the basic reproductive ratio, *R*_0_) defines the average number of secondary households infected as a consequence of infection in an average household during the early epidemic [6,33]. Although the transmission parameter is chosen such that *r* is the same in all models, those with household structure allow the amplification of infection within the household, which naturally produces a larger R*. The equilibrium level of infection, $\bar{I}$, is comparable between the four models, as for a fully immunizing infection prevalence is largely determined by the birth-rate generating new susceptibles. This birth rate provides an upper bound on the equilibrium incidence of infection, with lower values only occurring when individuals escape infection their entire lives. The prevalence is marginally higher for the age-structured model (compared to the other three) as this has greater transmission between school-age children (where the majority of the infection is maintained) but does not suffer from the within-household depletion of susceptible individuals. Finally, the probability of infection during childhood, *P*_*R*_, is calculated the chance of an individual being infected before they leave home and is increased slightly by the addition of either household or age structure which focuses infection into children, with a further increase when these two structures are combined.

Figure 3 shows distribution of cases per household (conditional on household size) for the four transmission models. In the homogeneous-mixing model, due to the lack of any structure, the probability of having at least one case increases (almost) linearly with the number of individuals; this idealized pattern is plotted as an open pink bar in the other models. It is the departure from this idealized linear case that informs about the actions of age and household structure. For the age-structured model, the greater mixing between school-children means that larger households (with more children) have a disproportionately higher risk of infection (the open circles correspond to the total expected amount of infection in households). However, for older households (red) which are likely to contain older children, the chance of infection is less than in the homogeneous model as there is a greater chance that these older children are immune from past infection. In both the homogeneous and age-structured model, the chance of observing multiple infections within the same household is vanishingly rare as within household transmission has been ignored. The household-structured model allows for local outbreaks such that multiple members of the same household are infected at the same time. This is visualised in the bottom two subplots of figure 3 using colour-coded stacked bars, with the height of each segment corresponding to the proportion of households containing a given number of cases. These local outbreaks lead to higher total levels of infection (as shown by the open circles) concentrated in the large households where sibling-to-sibling transmission is likely. However, household outbreaks are generally limited in scale due to levels of immunity in the household from historic infections. The full model, which includes both household and age structure, produces equilibrium distributions with a more pronounced difference between older and younger households than models with either one of these transmission structures in isolation. Large households in the earlier demographic stages (blue bars) are likely to contain school-age children who expose the household to age-structured transmission, while also containing younger children who lack previous exposure and so are susceptible to infection via these school-age siblings. While, in general, we expect age-structured mixing to boost infection (due to assortativity) and household-structured mixing to impede it (due to susceptible depletion), these results demonstrate that the two structures can act synergistically to amplify the concentration of infection into large young households.

### 3.3. Impact of disease and demography

Having gained an understanding of the effects of model structure, we now restrict our attention to the full model (with both age and household structure) and consider the impact of epidemiological and demographic parameters. The epidemiological parameters are those listed in table 1, such that measles has a greater rate of transmission across a contact, but a slightly shorter infectious period than mumps. UK- and Kenya-like demographic parameters are used as exemplars of a high-income country with relatively low population growth and a stationary population-age pyramid (similar numbers of individuals across most ages) and a low-income country with relatively high population growth and an expansive population-age pyramid (with higher numbers of younger ages). The main demographic differences between the UK and Kenya are captured by a much higher number of children per woman in Kenya (leading to a higher birth-rate) and a slightly shorter life expectancy; while Kenyan age-structured mixing is less assortative than the UK ([10,11], electronic supplementary material).

To illustrate the effects of epidemiological and demographic parameters on the full age- and household-structured model, we first calculated early growth dynamics and equilibrium distributions for measles- and mumps-like diseases in UK- and Kenya-like populations (table 3). As expected, we find that measles has greater growth than mumps in a naive population (as characterized by both *r* and R*) and that these measures of early behaviour are higher in Kenya than the UK. Such findings are attributable to the greater transmission rate of measles across a contact compared to mumps, and the larger average family size (and hence greater number of close contacts) in the Kenyan population compared to the UK. When considering endemic quantities, the higher birth-rate in the Kenya-like population (due to larger family sizes) is the main factor determining the endemic prevalence of infection ($\bar{I}$), with relatively little difference between measles and mumps. The population birth rate, and hence the rate that new susceptible individuals are produced, generates an upper bound on the equilibrium level of infection—lower values of equilibrium prevalence only occur when individuals escape infection for their entire lives. By contrast, the proportion of children infected before they leave home (*P*_*R*_) is strongly influenced by both the epidemiological characteristics and the demography of the population; almost all children are predicted to catch measles in a Kenyan-like population, but less than half predicted to catch mumps in a UK-like population.

**Table 3.** Early growth parameters, equilibrium prevalence, and childhood infection probability for measles- and mumps-like diseases in UK- and Kenya-like populations, from the household infectious disease model with demography. The corresponding household prevalence distributions are visualized in figure 4.

		*r*	R*	$\bar{I}{\times 10}^{4}$	*P*_*R*_
measles	UK	0.968	15.283	1.886	0.908
	Kenya	1.781	44.334	3.086	0.990
mumps	UK	0.133	2.691	1.760	0.480
	Kenya	0.320	6.728	3.083	0.833

**Figure 4.**
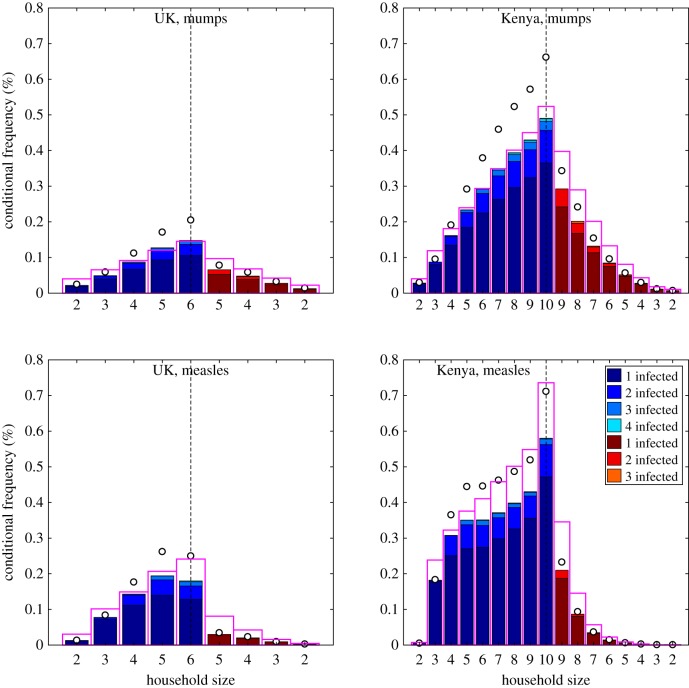
Percentage distribution of cases per household stratified by demographic state under UK- and Kenya-like demographic parameters for measles- and mumps-like infectious parameters. For purposes of clarity, we do not plot the percentage of disease-free households. As in figure 2, blue bars correspond to younger households (the first two rows in figure 1) and red to older households (the second two rows in figure 1). The pink open bars correspond to the results from the homogeneous mixing model with the same early growth rate (*r*) to demonstrate the effect of stratifying contact behaviour; the open circles show the total amount of infection in the households accounting for multiple infections. All plots are at the same scale to improve comparison, and we note that the data in figure4*a* is the same as that in figure 3*d*.

The histograms of infection levels in different household types provide more details of the underlying dynamical behaviour (figure 4, again the frequency of 1, 2, 3 or more cases conditional on the size and phase of the household is shown; the electronic supplementary material contains plots that show the absolute frequency, such that the lower density of the largest households is reflected). As before, the equilibrium distribution from a homogeneous model with the same early growth rate (pink bars) shows the simplest assumption where heterogeneities are only driven by the size and age of the household, households containing older individuals are already likely to have been infected experience less infection leading to nonlinear infection probability with household size. We compare our full model predictions to this homogeneous ideal. For mumps in both the UK and Kenya, the action of age and household structure is to exaggerate the differences between older and younger households, and between small and large households (*cf* pink bars and open circles)—due to the greater concentration of infection in school-age children, and the concentration of school-age children in larger younger households. For mumps in Kenya, we observe a slight saturating effect, with larger households less likely to experience infection than in the homogeneous model (*cf* pink and solid bars); although the scale of the outbreaks within the infected households more than compensates for this effect. When considering a highly transmissible infection like measles, this saturation is far greater as fewer children escape early infection and therefore reservoirs of susceptibility cannot build-up within households. In this way, young households of size 5 in the UK are predicted to contain more measles than households of size 6. In Kenya, these nonlinear saturating effects are even more pronounced: infection is concentrated in households with young children and these young households in general only ever have single measles cases; two or more cases within the household is relatively uncommon despite the strong rate of within-household transmission. This saturation is amplified by age and household structure, as age-structured transmission concentrates infection in school-aged children and household structure leads to rapid infection of any (younger) susceptible siblings. For measles-like infection in a Kenya-like population, households of size 10 show a substantially elevated level of infection in both the full and homogeneous model; this is because (under our demographic assumptions) such households always contain very young children who are the most likely to become infectious.

## 4. Discussion

The strong interactions within households and between individuals of similar ages dominates epidemiological transmission dynamics for a range of infectious diseases. The POLYMOD study [10] was revolutionary in quantifying our understanding of age-structured mixing, and shows strong diagonal and off-diagonal elements within the mixing matrix corresponding to connections between similar age cohorts and within families. This work has led to a number of diary-based studies that have improved our knowledge of age-structured mixing [11,34,35], and this wealth of new data has been pivotal in helping to produce accurate models of multiple infectious diseases [36,37]. However, in such models, there is no distinction between the repeated close-contact within the home environment and a ‘random’ contact; the repeated nature of contacts within the household means that this pool of susceptibles is rapidly depleted reducing the transmission potential compared to the frequently used homogeneous mixing assumption. By contrast, household models [6,13] explicitly recognize both the strong within-household transmission and the rapid depletion of susceptibles within the household environment, but generally do not capture the structure of between household mixing and ignore births into the population, restricting their application to epidemic scenarios [14,15]. Here, we have combined these two approaches to generate simple mechanistic models that capture the impact of household and age-structured mixing on the spread and distribution of endemic infectious diseases. A range of infectious and demographic settings can be simulated by choosing suitable parameters. While our model specifically describes infection in a population of nuclear households, our basic approach of coupling infectious and demographic dynamics can be applied to a more diverse set of demographic settings by coupling to a more complex set of demographic events.

Although high-dimensional, our model can be formulated from mechanistic principles and can be expressed as a series of coupled ODEs. These equations can be re-written in terms of matrix operations, with nonlinear transmission terms acting to re-scale elements of these matrices; this allows us to exploit computationally efficient methods to calculate early growth rates of outbreaks as well as endemic equilibria. The emergent dynamics are highly complex and are the result of four interacting processes: (i) age structure acts to focus transmission within school-age children (due to their greater social mixing) which in turn disperses infection between households; (ii) in contrast with age structure, household structure concentrates infection within family groups and hence leads to transmission between distinct age classes; (iii) exposure over time means that an individual’s probability of immunity increases with age so that older households tend to contain a higher density of immune individuals and thus experience less infection, (iv) the continuous circulation means that newborns often find themselves in households which have already experienced infection, increasing the local density of immune individuals and sheltering newborns from infection, making large local outbreaks relatively rare. The tension between these four factors is determined by the demographic and epidemiological parameters. Our comparison between model structures includes the important finding that while age structure and household structure have opposite effects on the population-level growth of infection, they act in tandem to concentrate infection in younger households. This demonstrates the importance of more detailed outputs in understanding the behaviour of complex models. From a public-health perspective our results suggest that household-based control (such as prophylaxis [14] or cocoon vaccination [38,39]) is most likely to be effective for weakly transmitted or non-immunizing infections where many older individuals remain susceptible and hence household outbreaks are common.

Although our model incorporates only a single level of spatial structure (that of the household), it can easily be adapted to incorporate more detailed location-based contact data. Contact studies including the POLYMOD study stratify contacts by location according to categories including school, work and transport [10], allowing us to express our age-structured contact matrix as a sum of location-based components. By considering this extra level of structure, we can then model interventions such as school closure by scaling down or removing the appropriate contact rates.

## Supplementary Material

Supplementary Information

## Data accessibility

All of the code used to create the tables and figures in this paper and its supplement is available at github.com/JBHilton/HiltonKeeling_EndemicDiseases.
