# Supplementary material for: Incorporating household structure and demography into models of endemic disease
Source: J R Soc Interface. 2019 Aug 7;16(157):20190317. doi: 10.1098/rsif.2019.0317 (PMC6731502; doi:10.1098/rsif.2019.0317)
Supplement: Supplementary Information [file rsif20190317supp1.pdf]

Supplementary information for *Incorporating household structure and demography into models of endemic disease*, accepted for publication in Journal of the Royal Society Interface

Joe Hilton[a,b] and Matt J. Keeling[b,c,d,1]

[a]MathSys CDT, Mathematics Institute, University of Warwick, Coventry, CV4 7AL, UK
[b]Zeeman Institute (SBIDER), University of Warwick, Coventry, CV4 7AL, UK
[c]Mathematics Institute, University of Warwick, Coventry, CV4 7AL, UK
[d]School of Life Sciences, University of Warwick, Coventry, CV4 7AL, UK

1 Introduction

The household infectious disease model with demography combines a Markov chain model for the evolution of a household with a Markovian SIR transmission model that incorporates internal mixing within the household, homogeneous between-household mixing, and age-structured mixing, also on the between-household scale. As such there are a large number of parameters (Table S1) governing the demographics, epidemiology and status of a household.

In this supplement we provide a detailed account of the model's definition and analysis. In Section 2 we explain how to calculate model outputs including the early growth rate and equilibrium prevalence. In Section 3 we perform sensitivity analysis on the parameters τ and σ. In Section 4 we give a detailed description of the model structure, and Section 5 explains how to incorporate demographic and contact survey data into this model. Finally, in Section 6 we detail the calculation of the age structured infection rate λ_T.

All of the code used to create the tables and figures in the paper and this supplement is available at `github.com/JBHilton/HiltonKeeling_EndemicDiseases`.

2 Calculation of epidemiological quantities

The model we construct in Section 4 is high-dimensional; for $N = 5$, and using the k values in Table 2 there are 1170 possible household states. We therefore need to compute some simple aggregate quantities that inform about the important epidemiological behaviours:

2.1 Disease-free equilibrium and household size distribution

The system of ODEs

$$\frac{\mathrm{d}\underline{H}}{\mathrm{d}t} = \underline{H}\boldsymbol{Q}_{\mathrm{Demo}}(\underline{H}) \tag{1}$$

defines the evolution of the demographic class distribution. In the disease-free setting all individuals remain susceptible, fixing P_R at zero, and so in this setting $\boldsymbol{Q}_{\mathrm{Demo}}$ is constant. The dynamics are then determined by the linear system

$$\frac{\mathrm{d}\underline{H}}{\mathrm{d}t} = \underline{H}\boldsymbol{Q}_{\mathrm{Demo}}(P_R = 0) \tag{2}$$

**Quantity**	**Meaning**
N	Household size
$N_{\max}$	Maximum household size
k	Current counter state
$T = T(N, k)$	Current demographic class
k_B	Number of ticks between births
k_L	Number of ticks between last birth and first leaving
k_R	Number of ticks between last child leaving and reset
T_B	Mean between-birth interval
T_L	Mean age of child leaving home
T_D	Life expectancy
T_R	Mean interval between last child leaving and reset
τ	Unit time transmission rate
γ	Recovery rate
d_C	Mean duration of contacts between children of same household
d_{int}	Mean time per day spent exposed to internal contacts
d_{ext}	Mean time per day spent exposed to external contacts
$\boldsymbol{D}_{\text{ext}}$	Age-structured profile of external contacts
$\boldsymbol{D}_{\text{all}}$	Age-structured profile of all contacts
$\beta_{\text{int}}(= \tau d_{\text{int}})$	Internal transmission rate
$\beta_{\text{ext}}(= \tau d_{\text{ext}})$	External unstructured transmission rate
λ_T	External age-structured force of infection on individuals in a household in demographic class $T(N, k)$
σ	Convexity parameter measuring proportion of structured transmission
$\{C_1, ..., C_K\}$	Set of age classes used for age-structured mixing
$E_{T,i}$	Expected number of individuals in age class C_i in a household in state T
$\tilde{E}_{T,i}$	Conditional probability that an individual in age class C_i belongs to a household in state T
D_{ij}	Mean daily exposure time of age class C_i individuals to age class C_j individuals
H_T	Population-level proportion of households in demographic class T
$\boldsymbol{Q}$	Markov chain transition matrix
$\boldsymbol{Q}_{\text{Demo}}$	Transition matrix associated with demographic events
$\boldsymbol{Q}_{\text{Int}}$	Transition matrix associated with internal infection events
$\boldsymbol{Q}_{\text{Ext}}$	Transition matrix associated with external infection events
$\underline{H}$	Distribution of system states
$\underline{H}^*$	Equilibrium state distribution
$\underline{H}_{I=0}$	Disease-free equilibrium distribution
r	Early infectious growth rate
R^*	Household-level reproductive ratio
$\bar{I}$	Population-level infectious prevalence
$\bar{I}_T$	Infectious prevalence across households in demographic class T
$\bar{I}^i$	Infectious prevalence within age class C_i
P_R	Probability of infection occurring before leaving home

Table 1: Complete list of parameters and outputs associated with the household infectious disease model with demography.

The disease-free equilibrium $\underline{H}_{I=0}$ distribution is the eigenvector associated with the dominant (zero) eigenvalue of this system [6]. The only states with nonzero probability are those with $S = N$, so that confining our attention to these states gives us the equilibrium joint distribution of (N, k). This provides a ready means of calculating the equilibrium distribution of demographic classes, since the probability H_T that a household is in demographic class T is given by $H_T = \underline{H}_{I=0}(N(T), 0, 0, T)$. This distribution of demographic classes is independent of the infectious status of the system, so that the marginal distribution with respect to infectious status is always the same as that of $\underline{H}_{I=0}$. The disease-free equilibrium $\underline{H}_{I=0}$ is used as the basis for the calculation of early growth behaviour.

2.2 Endemic equilibrium

Following the procedure described by Ross et. al.[15], the equilibrium distribution is calculated by iteratively converging on a distribution $\underline{H}^*$ such that the dominant eigenvalue of $\boldsymbol{Q}(\underline{H}^*) = \boldsymbol{Q}_{\text{Demo}}(\underline{H}^*)+\boldsymbol{Q}_{\text{Int}}+\boldsymbol{Q}_{\text{Ext}}(\underline{H}^*)$ (by definition this leading eigenvalue is zero) has leading eigenvector $\underline{H}^*$.

The external transmission rate matrix $\boldsymbol{Q}_{\text{Ext}}$ depends on $\underline{H}$ via the variables $\bar{I}$, $\bar{I}_T$, while $\boldsymbol{Q}_{\text{Demo}}$ depends on P_R; hence $\boldsymbol{Q}$ and in turn its dominant eigenvector depends on these three variables. We therefore initialise these three variables so that $P_R^0 = 0$, $\bar{I}^0 = \bar{I}_T^0 \ll 1$, and calculate the associated transition matrix $\boldsymbol{Q}^0$; the dominant eigenvector of this matrix is labelled $\underline{H}^1$. The probability distribution $\underline{H}^1$ defines values P_R^1, $\bar{I}^1$ and $\bar{I}_T^1$, which in turn define a transition matrix $\boldsymbol{Q}^1$. This leads us to define an iterative process such that $\underline{H}^{n+1}$ is the dominant eigenvector of $\boldsymbol{Q}^n$. We observe that this process rapidly converges on the population level equilibrium $\underline{H}^*$ and associated matrix $\boldsymbol{Q}$, and offers a robust and reliable means of finding the equilibrium of this high-dimensional non-linear system of ODEs.

The population-level prevalence is given by

$$\bar{I} = \frac{\sum\limits_{S,I,R,T} \underline{H}_{S,I,R,T} I}{\sum\limits_{S,I,R,T} \underline{H}_{S,I,R,T} N(T)} \tag{3}$$

and the demographic class-stratified prevalence is given by

$$\bar{I}_T = \frac{\sum\limits_{S,I,R} \underline{H}_{S,I,R,T} I}{\sum\limits_{S,I,R} \underline{H}_{S,I,R,T} N(T)}. \tag{4}$$

where the subscripts relate to a particular element of the vector $\underline{H}$.

One way to visualise the equilibrium behaviour is by plotting the distribution of cases per household, stratified by household demographic class. Figures 3 and 4 of the main paper show bar charts of the probabilities of I cases appearing in a household of size N with demographic counter from 1 to $k_B + k_L$ and $k_B + k_L + 1$ to $2k_B + k_L + k_R$, corresponding respectively to the first two phases and last two phases of the demographic model. The probability that a household of size N with $1 \leq k \leq k_B + k_L$ contains exactly I infectious cases is given by

$$\frac{\sum\limits_{T \in Z^N_{Early}} \sum\limits_{S+R=N-I} \underline{H}_{S,I,R,T}}{\sum\limits_{T \in Z^N_{\text{Early}}} \sum\limits_{S,I,R} \underline{H}_{S,I,R,T}} \quad \text{where } Z^N_{\text{Early}} = \{T : 1 \leq k(T) \leq k_B + k_L, N(T) = N\}, \tag{5}$$

whilst the equivalent probabilities for households with $k_B + k_L + 1 \leq k \leq 2k_B + k_L + k_R$ is

$$\frac{\sum\limits_{T \in Z^N_{\text{Late}}} \sum\limits_{S+R=N-I} \underline{H}_{S,I,R,T}}{\sum\limits_{T \in Z^N_{\text{Late}}} \sum\limits_{S,I,R} \underline{H}_{S,I,R,T}} \quad \text{where } Z^N_{\text{Late}} = \{T : k_B + k_L + 1 \leq k(T) \leq 2k_B + k_L + k_R, N(T) = N\}. \tag{6}$$

The probabilities in Equations (5) and (6) are conditioned on demographic class. As well as the probability that a household of size N in the early (or late) demographic phases contains I infectious cases, it can also be useful to plot the the proportion of households which are in the early (respectively late) demographic phases and are of size N and which contain I infectious cases. This is done by removing the conditioning, resulting in the respective formulae

$$\sum_{T \in Z^N_{\text{Early}}} \sum_{S+R=N-I} \underline{H}_{S,I,R,T} \tag{7}$$

and

$$\sum_{T \in Z^N_{\text{Late}}} \sum_{S+R=N-I} \underline{H}_{S,I,R,T}. \tag{8}$$

The probability distributions defined by Equations (5) and (6) describe the burden of disease on households of given sizes and ages, whereas the proportions defined by Equations (7) and (8) can be interpreted as indicating the contribution of each household type to the population-level infectious presence, taking into account the fact that high disease burdens in rare household types may not contribute much to population-level transmission. These proportions are plotted in Figures 1 and 2. The bar charts in Figure 1 indicate that for mumps-like infectious parameters older households without children make large contributions to the population-level prevalence under all transmission structures, despite infection being comparably rare within these households. However, Figures 2a and 2b indicate that this is not the case for measles, where the fast spread of infection means that most adults have already been exposed to infection before establishing their own household.

Age structured mixing is defined in terms of a set of discrete age classes $C_1, ..., C_K$, which are listed for our UK- and Kenya-like populations in Table 4. The age-structured transmission process is detailed in Sections 4.1 and 6 and in its implementation we calculate the expected prevalence within the household of an individual of age class C_i. This gives an indication of each age class's exposure to household infection and their contribution to the population-level age-structured transmission dynamics. The prevalences give us a further visualisation of the model's equilibrium behaviour and are plotted as bar charts in Figures 3 and 4. Figure 3 suggests that moving between different transmission pathways has a limited impact on the distribution of infection. Figure 4 indicates that Kenya's burden of disease is more skewed towards younger age classes than that of the UK, implying a younger age at first infection. The "elder" age class C_6 in the Kenya-like population corresponds precisely to members of two-person households whose children have left home (provided they had children in the first place), so that the relatively high prevalence associated with age class C_6 indicates a relatively high burden of disease in these older households.

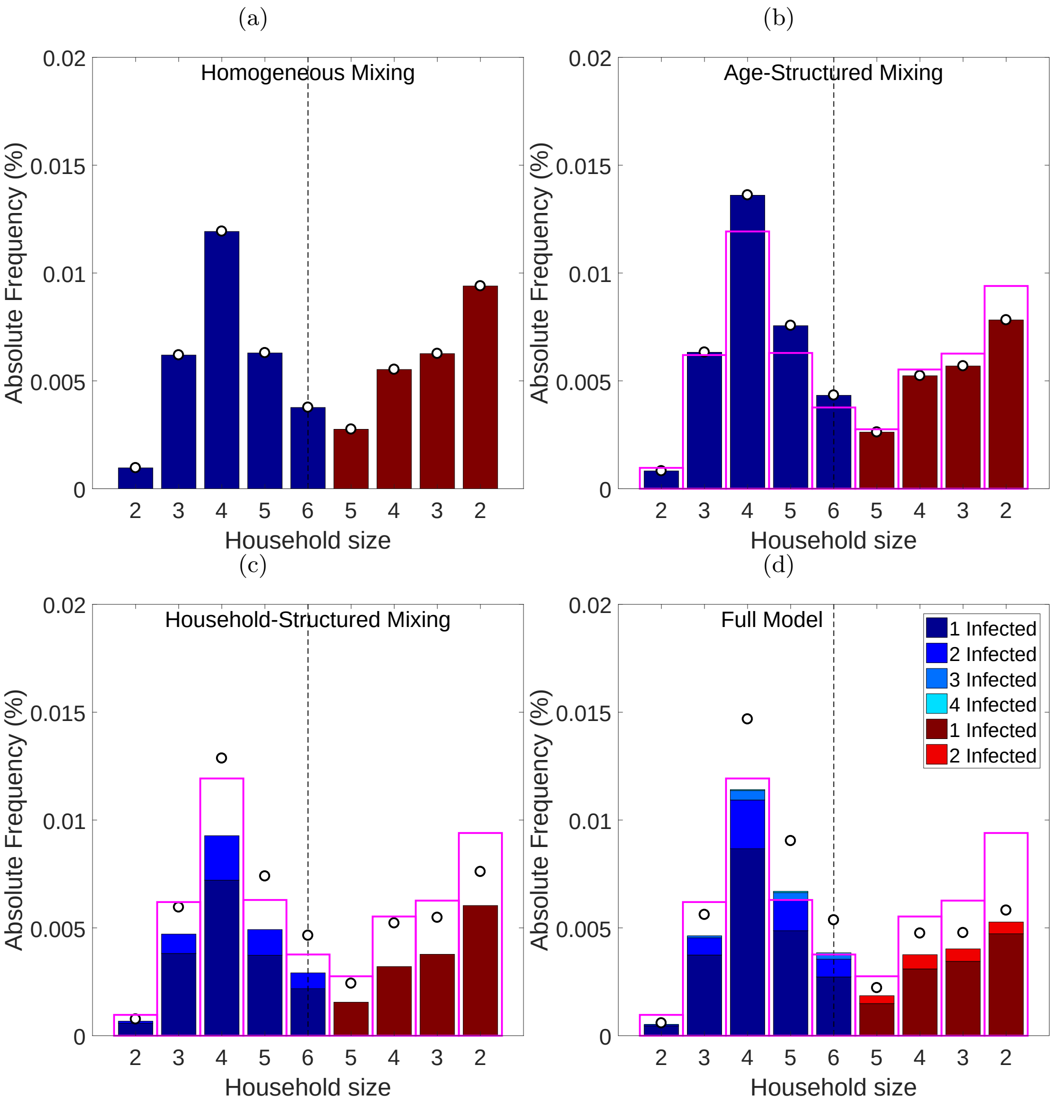

Figure 1: Percentage distribution of cases per household stratified by the size (and demographic status – young / old) of the household, for the four different models. The distributions are not conditioned on household size, so that the size of each bar corresponds to the absolute proportion of households in a given aggregation of demographic-infectious states. This should be compared to Figure 3 of the main paper which presents the equivalent distributions conditioned on household size. Blue bars correspond to the first two phases of the demographic process (prior to the eldest child leaving home), red bars correspond to the third and fourth phases (after the eldest child has left). The pink open bars correspond to the results from the random-mixing model, which are shown for ease of comparison; the open circles show the total amount of infection in the households accounting for multiple infections.

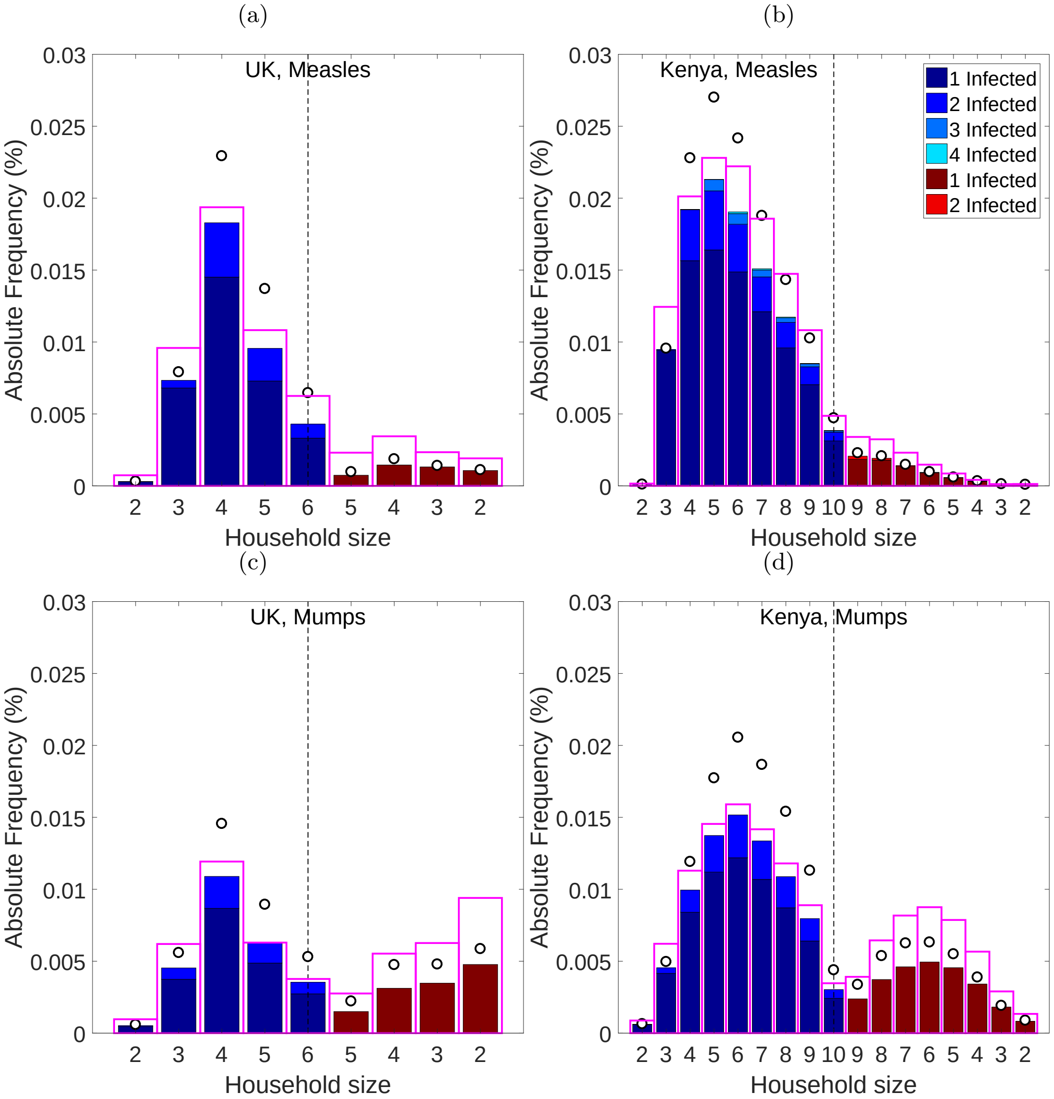

Figure 2: Percentage distribution of cases per household stratified by demographic class under UK- and Kenya-like demographic parameters for measles and mumps-like infectious parameters.The distributions are not conditioned on household size, so that the size of each bar corresponds to the absolute proportion of households in a given aggregation of demographic-infectious states. This should be compared to Figure 4 of the main paper which presents the equivalent distributions conditioned on household size. Blue bars correspond to the first two phases of the demographic process (prior to the eldest child leaving home), red bars correspond to the third and fourth phases (after the eldest child has left). The open circles show the total amount of infection in the households accounting for multiple infections.

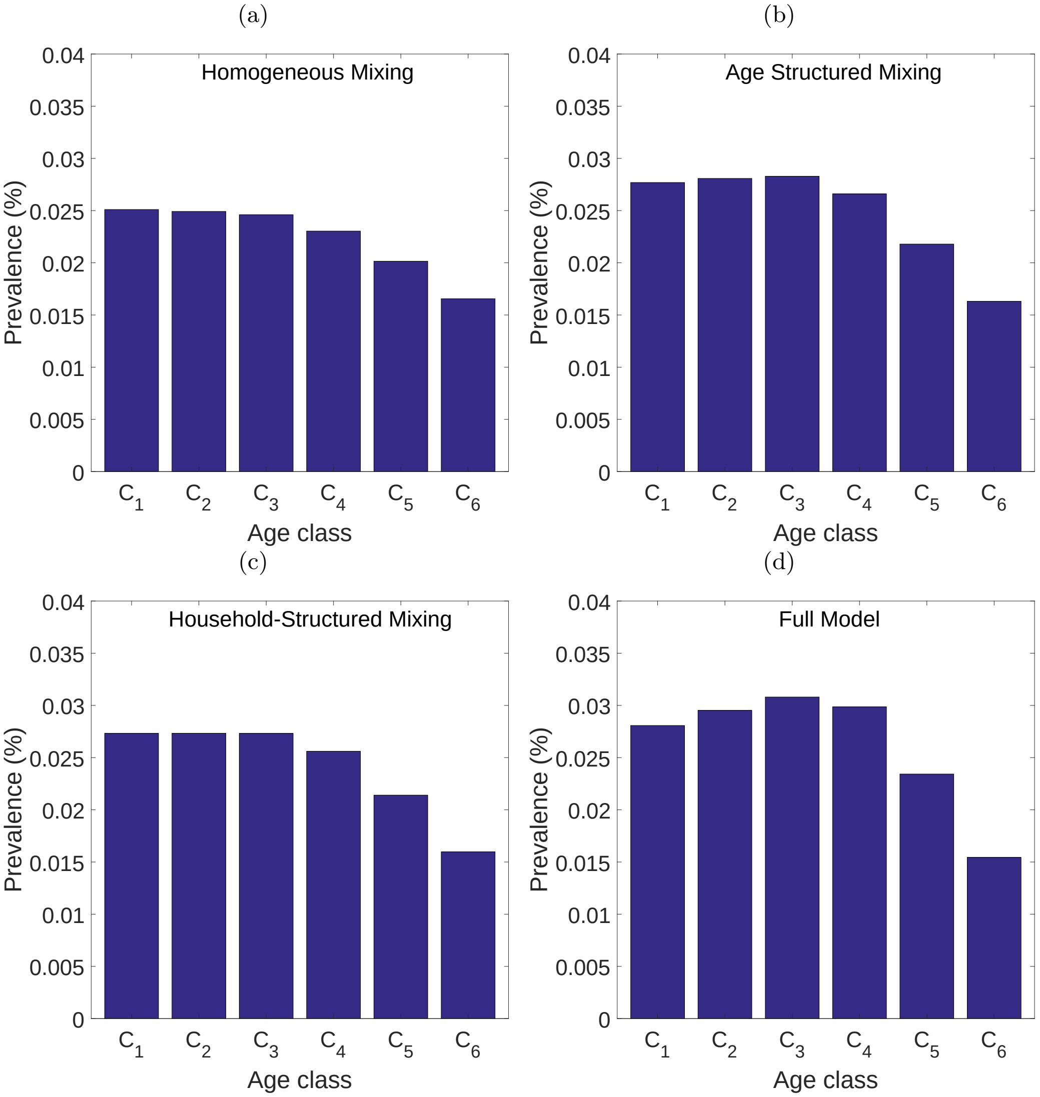

Figure 3: Equilibrium infectious prevalence by age class in the four transmission models, using parameters for the UK and mumps. The bars show the mean prevalence level across all household types that contain at least one individual of type C_i; as such this illustrates how households with younger individuals are most likely to be infected. Here the six age-classes are: C_1 under 1, C_2 1-2 years old, C_3 3-5 years old, C_4 6-10 years old, C_5 11-16 years old, C_6 17 or over; where ages are rounded down to the whole year.

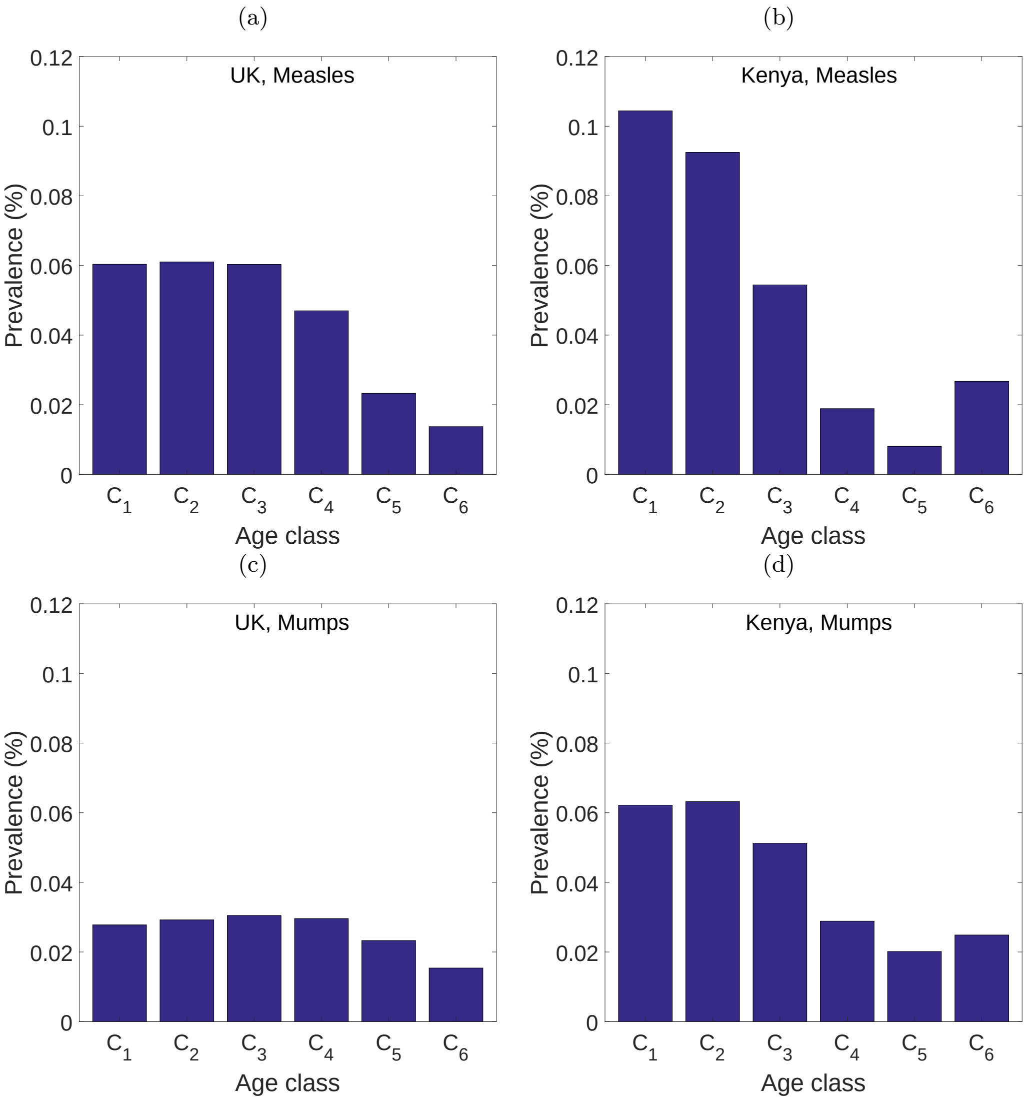

Figure 4: Equilibrium infectious prevalence by age class under UK- and Kenya-like demographic parameters for measles and mumps-like infectious parameters. The bars show the mean prevalence level across all household types that contain at least one individual of type C_i. We note that the six age-classes differ for the UK and Kenyan population. For the UK: C_1 under 1, C_2 1-2 years old, C_3 3-5 years old, C_4 6-10 years old, C_5 11-16 years old, C_6 17 or over; while for Kenya: C_1 under 1, C_2 1-5 years old, C_3 6-15 years old, C_4 16-19 years old, C_5 20-50 years old, C_6 51 or over; here all ages are rounded down to the whole year.

2.3 Early growth rate r

The early exponential growth rate of the infection, r, is found by integrating forward the short time-scale dynamics

$$\frac{\mathrm{d}\underline{H}}{\mathrm{d}t} = \underline{H}\boldsymbol{Q}(\underline{H}) = \underline{H}(\boldsymbol{Q}_{\mathrm{Int}} + \boldsymbol{Q}_{\mathrm{Ext}}(\underline{H})).$$

starting close to the disease-free equilibrium $\underline{H}_{I=0}$. This process can be achieved with far greater efficiency by linearising about this disease-free equilibrium, assuming that the number of households that contain infectious or recovered individuals is small (order ϵ) during this early growth phase. This linearisation is equivalent to assuming that only totally susceptible households get infected by external processes (the chance of an already infected household getting infected for a second time is order ϵ^2 and can be ignored). The linear equations can be solved by an eigenvalue approach, with the leading eigenvalue giving the early exponential growth rate r and the associate eigenvector $\underline{H}_Q$ giving the quasi-equilibrium distribution of infection across household types.

2.4 Household level reproduction ratio R^*

The household level reproduction ratio R^* is defined as the expected number of new household outbreaks initiated by an averge household outbreak in a pristine population, or in other words the total number of successful external transmissions made by the members of an average household during an outbreak in that household. In a household-structured epidemic, R^* is an important threshold parameter in the sense that global epidemics are possible only if R^* exceeds one [2, 3]. Our calculation method is equivalent to the path integral formula for R^* used by Ross et. al. [15], generalised to a disease with multiple transmission pathways.

From the dominant eigenvector $\underline{H}_Q$ of the linearised system, we are able to calculate both the mean level of infection ($\bar{I}$) and the structured infection rate (λ_T). Together with the disease-free equilibrium, these early invasion parameters allow us to define the rate that new households are infected:

$$\text{Rate of Infection, } \underline{\lambda} = \underline{H}_{I=0}\boldsymbol{Q}_{\mathrm{Ext}}(\bar{I}, \lambda_T)$$

which is proportional to the distribution of newly infected household $\underline{H}_{\mathrm{new}}$, with the proportionally constant defined such that this distribution sums to one. We now define separate equations for the evolution of households and the generation of external infection:

$$\frac{\mathrm{d}\underline{H}}{\mathrm{d}t} = \underline{H}\boldsymbol{Q}_{\mathrm{Demo}}(P_R = 0) + \underline{H}\boldsymbol{Q}_{\mathrm{Int}} \approx \underline{H}\boldsymbol{Q}_{\mathrm{Int}} \tag{9}$$

and

$$\frac{\mathrm{d}\underline{R}}{\mathrm{d}t} = \underline{H}_{I=0}\boldsymbol{Q}_{\mathrm{Ext}}(\underline{H}) \tag{10}$$

with the starting conditions $\underline{H} = \underline{H}_{\mathrm{new}}$ and $\underline{R} = 0$. The sum over all components of the vector $\underline{R}$ gives the number of externally infected households by an average newly infected household, and in the long-term this sum has limit R^*. Since the size of the within-household outbreak is bounded by household size N, the probability of the outbreak ending will quickly converge towards 1 after around N/γ units of time, and hence the value of R^* can also be computed by integrating equations (9) and (10) over such time-scales.

2.5 Childhood infection probability P_R at equilibrium

The probability P_R of an individual being infected during childhood and thus being recovered when they start a new household is determined by the distribution of household infectious profiles at the end of the second and third phases of the demographic process. Denote by P_R' the probability that the parents of the household have infectious status R when the household is initiated at $k = 0$. When the counter reaches $k = k_B + k_L$ or $k = 2k_B + k_L$, a child is removed from the household, with an infectious status chosen

according to the infectious status of the household, (S, I, R). Since the household contains an expected $2P'_R$ individuals who were recovered at the start of the household's lifetime, we remove these individuals from our consideration, so that the child leaving the household is chosen from a pool of $S + I + R - 2P'_R$ individuals of whom $R - 2P'_R$ are in the recovered class. This gives rise to the formula

$$P_R = \frac{1}{\sum_{T \in Z_{\text{Leave}}} \underline{H}_{S,I,R,T}} \sum_{T \in Z_{\text{Leave}}} \sum_{S,I,R} \underline{H}_{S,I,R,T} \frac{R - 2P'_R}{N - 2P'_R} \quad \text{where } Z_{\text{Leave}} = T : k(T) = k_B + k_L \text{ or } k(T) = 2k_B + k_L. \tag{11}$$

Equation 11 defines P_R based on its "last generation" value P'_R. At endemic equilibrium P_R is constant across generations, and its value is found by performing the endemic equilibrium calculation detailed in Section 2.2, using Equation 11 to update P_R at each step.

3 Parameter sensitivity

The household infectious disease model is a Markov process with a large number of states and many parameters. We conducted some basic sensitivity analysis by calculating the behaviour of the epidemiological quantities defined in Section 2 as functions of transmission rate τ and σ, a tuning parameter which controls the degree of age-structure in external transmission. External transmission occurs as a convex combination of a structured and an unstructured term, with homogeneous external transmission at $\sigma = 0$ and fully age-structured external transmission at $\sigma = 1$. By choosing specific transmission parameters along with these extreme values of σ, the transmission component of our model can be reduced to homogeneous, purely age-structured, and purely household-structured dynamics, as is explained in Section 4.3. Without household structure, incrementing between $\sigma = 0$ and $\sigma = 1$ tunes the model from homogeneous to purely age-structured mixing, whereas with household structure, it tunes from the purely household-structured transmission model to the fully structured household infectious disease model with demography.

Dependence on τ is plotted in Figures 5 to 8. To gain a broad understanding of behaviour, we calculated dependence in UK- and Kenya-like settings for infectious periods of 7 and 14 days, under all four transmission models. The value of τ ranged from 0.1 to 2. The procedure for estimating τ defined in Section 5 gives $\tau = 0.412$ for mumps and $\tau = 1.782$ for measles, meaning our analysis ranges between low and high levels of transmissibility. The early growth parameters r and R^* are approximately linear in τ, as is to be expected since transmission intensifies with increasing τ. Figure 5 demonstrates that early growth is consistently slowed when contacts are structured by household, but that age structure appears to have minimal effect. Figure 6 indicates that age structure boosts household-to-household transmission since the full model with both age and household structure produces higher values of R_* than the purely household-structured model. We note here that in the absence of household structure (i.e. for the homogeneous and purely age-structured model), R_* is equal to the basic reproductive number R_0. The plots in Figure 7 suggest that although Equilibrium prevalence $\bar{I}$ increases sharply with smaller values of τ, transmission is limited by demographic constraints. Comparing the results for $\gamma^{-1} = 7$ and $\gamma^{-1} = 14$ demonstrates the intuitive result that doubling the infectious period doubles this demographically-imposed maximum prevalence. Childhood infection probability P_R, plotted in Figure 8, increases towards 1 with τ. Infection during childhood is consistently most likely under purely age-structured mixing, which facilitates the spread of infection specifically between households containing young children, and least likely under purely household-structured mixing, which impedes between-household transmission.

Dependence on σ is plotted in Figures 9 to 12 for measles- and mumps-like transmission parameters in UK- and Kenya-like demographic settings, in the absence and presence of household-structured mixing. Dependence of the early growth parameters on σ is extremely weak, although under measles-like parameters there is a noticeable increase in R_* in the full age- and household-structured model. As in Figure 6, the curves for the model without household structure correspond to basic reproductive ratio R_0. The dependence of prevalence on σ, plotted in Figure 11, suggest that stratifying transmission by household consistently decreases prevalence, although only by a small amount (at most a few cases per hundred thousand people). The details of the relationship between $\bar{I}$ and σ are specific to the population-disease setting in question,

although differences in transmission structure appear to be more relevant for mumps. Figure 12 demonstrates that increased age-structured mixing increases the probability of exposure to disease during childhood in all demographic and infectious settings.

Taken together, the results of the sensitivity analysis indicate that the early growth parameters are smoothly dependent on the transmission parameters. The equilibrium behaviour displays complex dependence on transmission parameters, but is qualitatively similar across the two demographic settings we have studied. This in turn suggests that model behaviour is robust with respect to the demographic parameters.

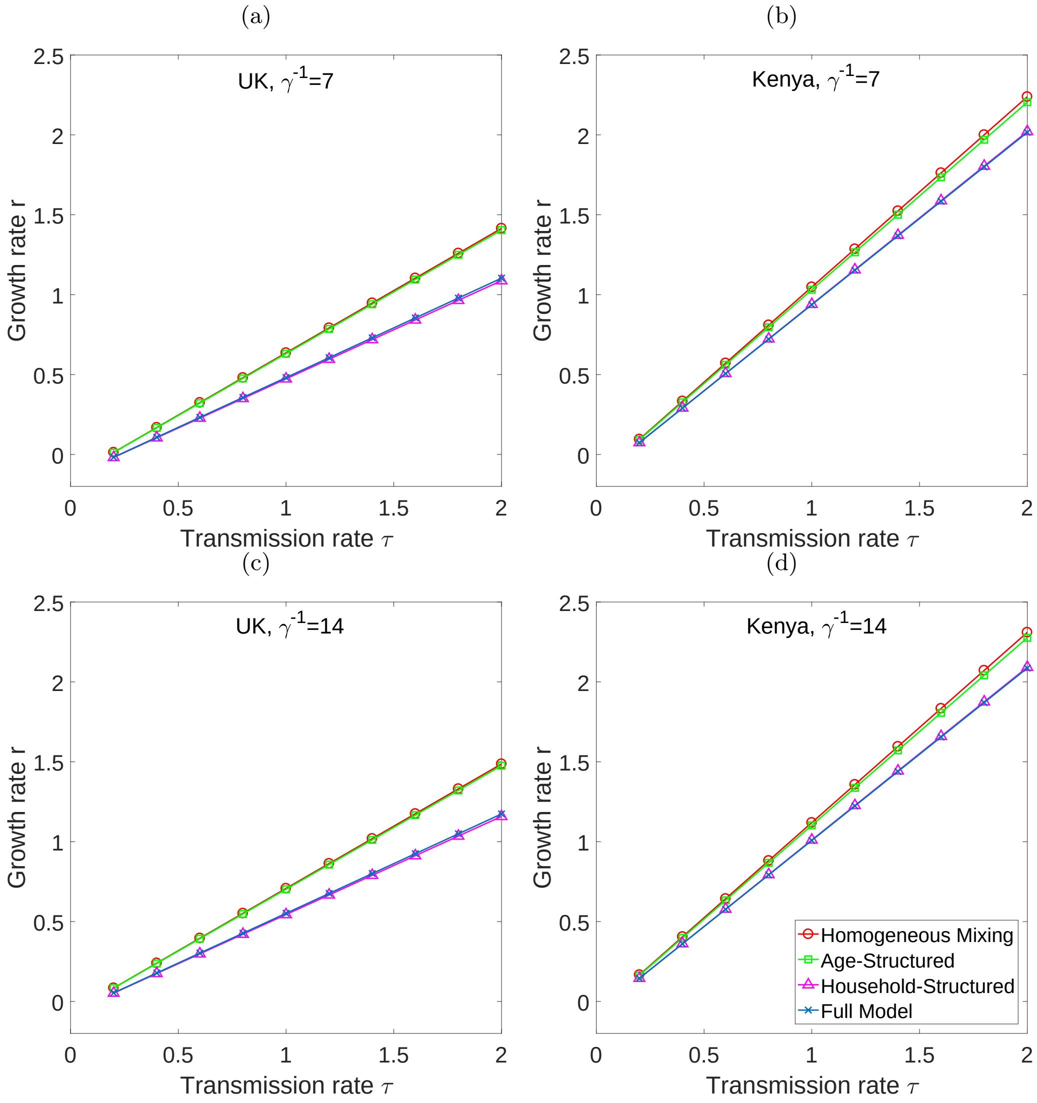

Figure 5: Early growth rate r as a function of transmission rate τ across a contact. Results are shown for UK- and Kenya-like demographic parameters, and for average infectious periods of 7 and 14 days. We note that the the growth rate increases close to linearly with increasing transmission rate.

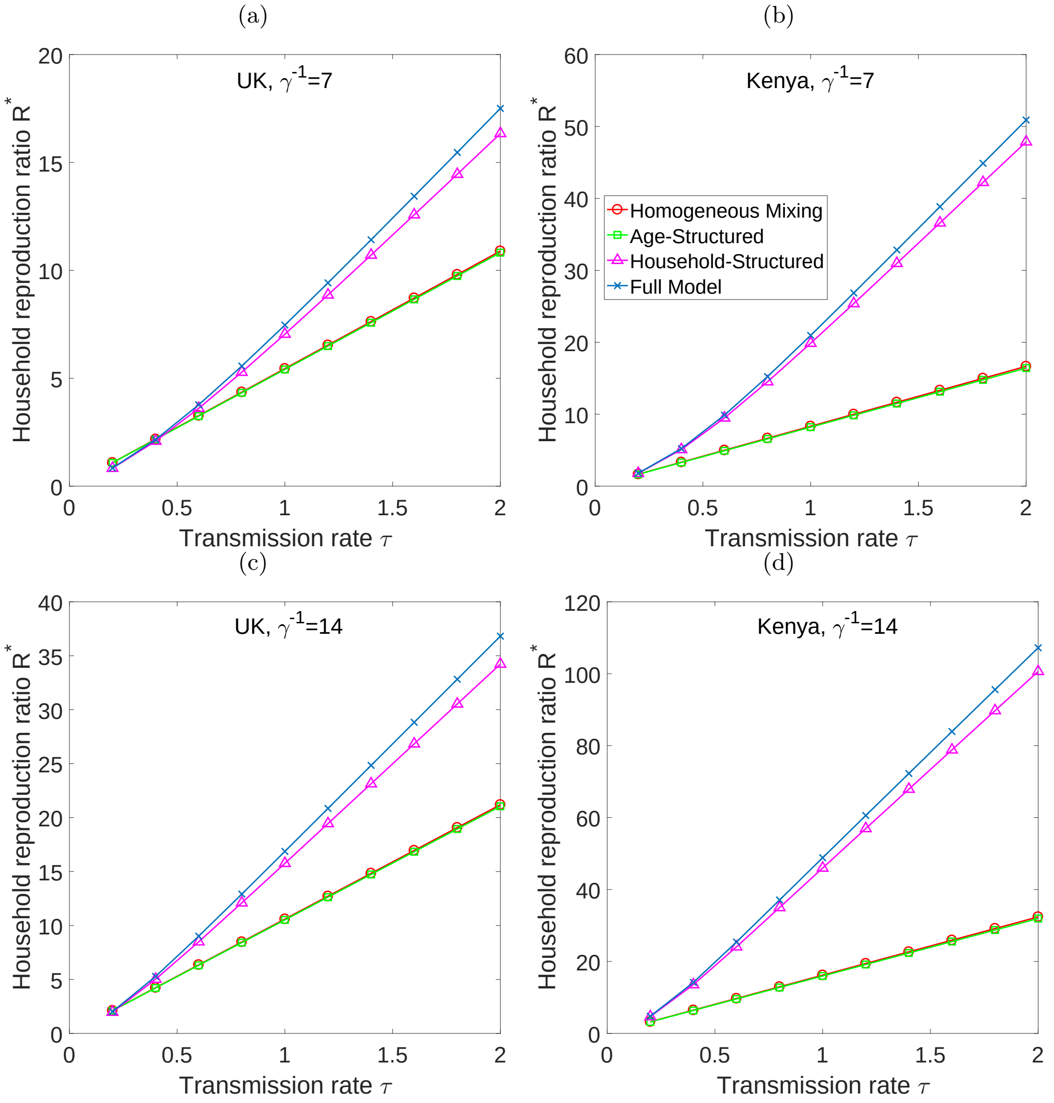

Figure 6: Household-level reproductive ratio R^* as a function of transmission rate τ across a contact. Results are shown for UK- and Kenya-like demographic parameters, and for average infectious periods of 7 and 14 days. There is clear non-linear behaviour for models with household structure due to the amplification of early infection within the household.

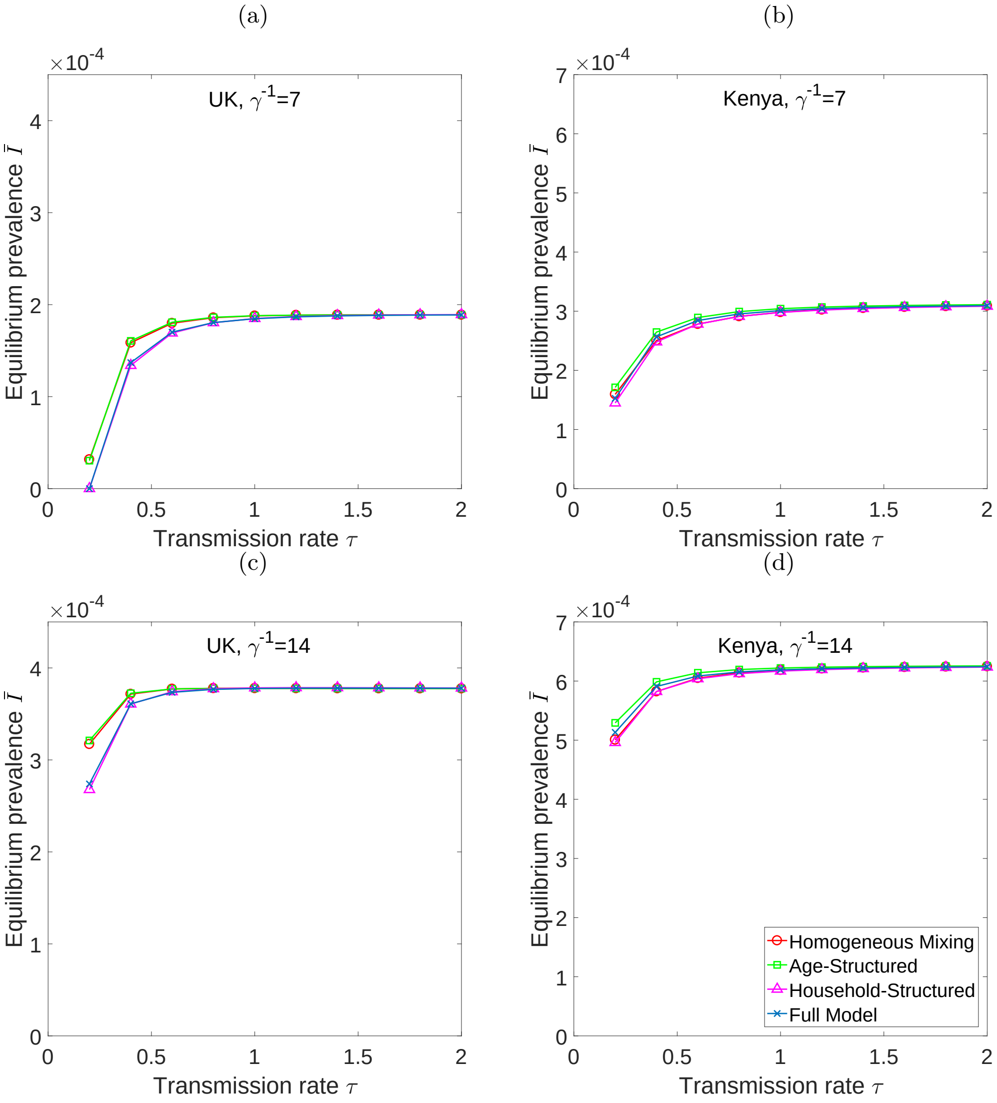

Figure 7: Equilibrium infectious prevalence $\bar{I}$ as a function of transmission rate τ across a contact. Results are shown for UK- and Kenya-like demographic parameters, and for average infectious periods of 7 and 14 days. The prevalence rapidly asymptotes with increasing transmission, only in low transmission setting do individuals escape infection.

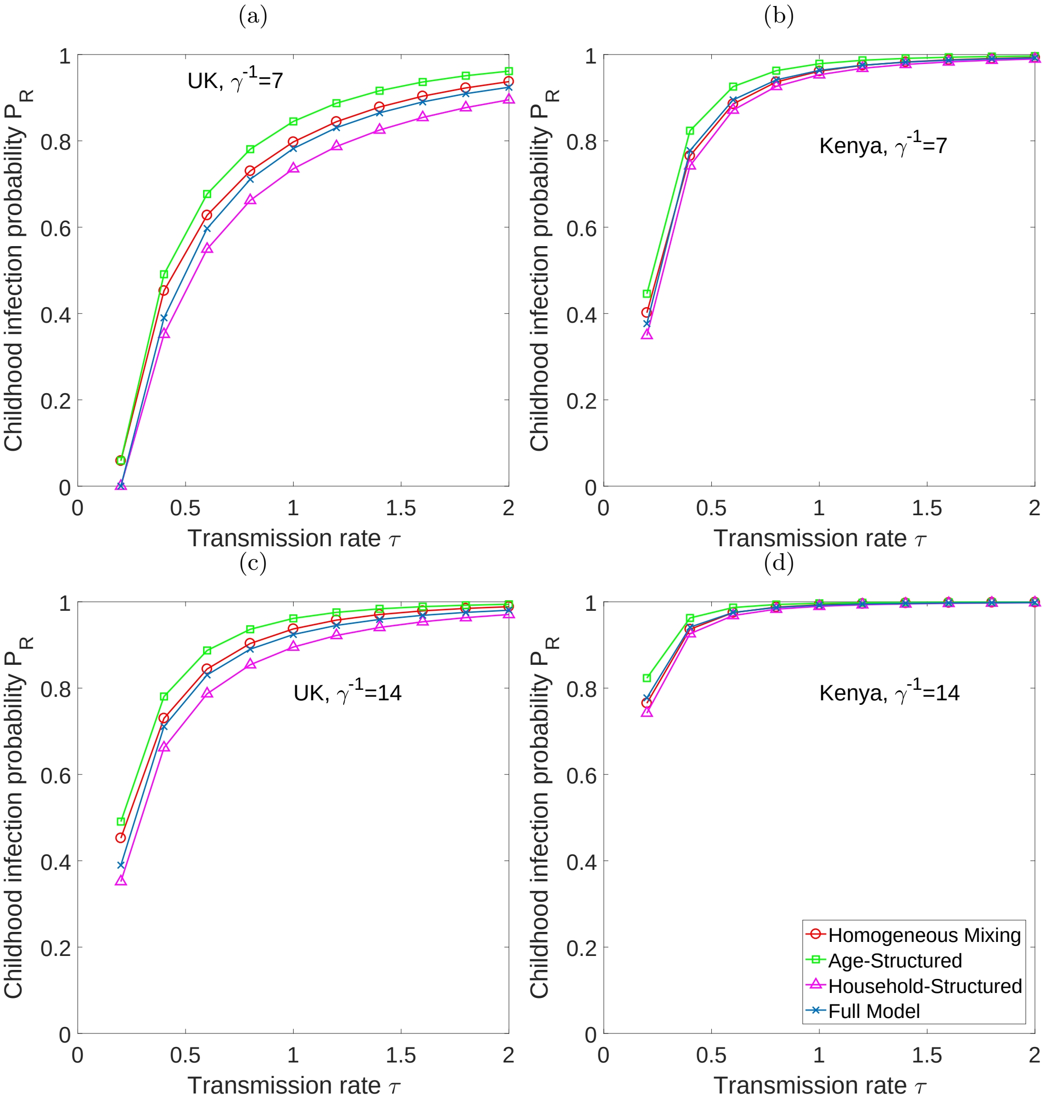

Figure 8: Endemic childhood infection probability P_R as a function of transmission rate τ across a contact. Results are shown for UK- and Kenya-like demographic parameters, and for average infectious periods of 7 and 14 days. Due to the different population structure, results for the UK require higher rates of transmission before reaching their asymptote.

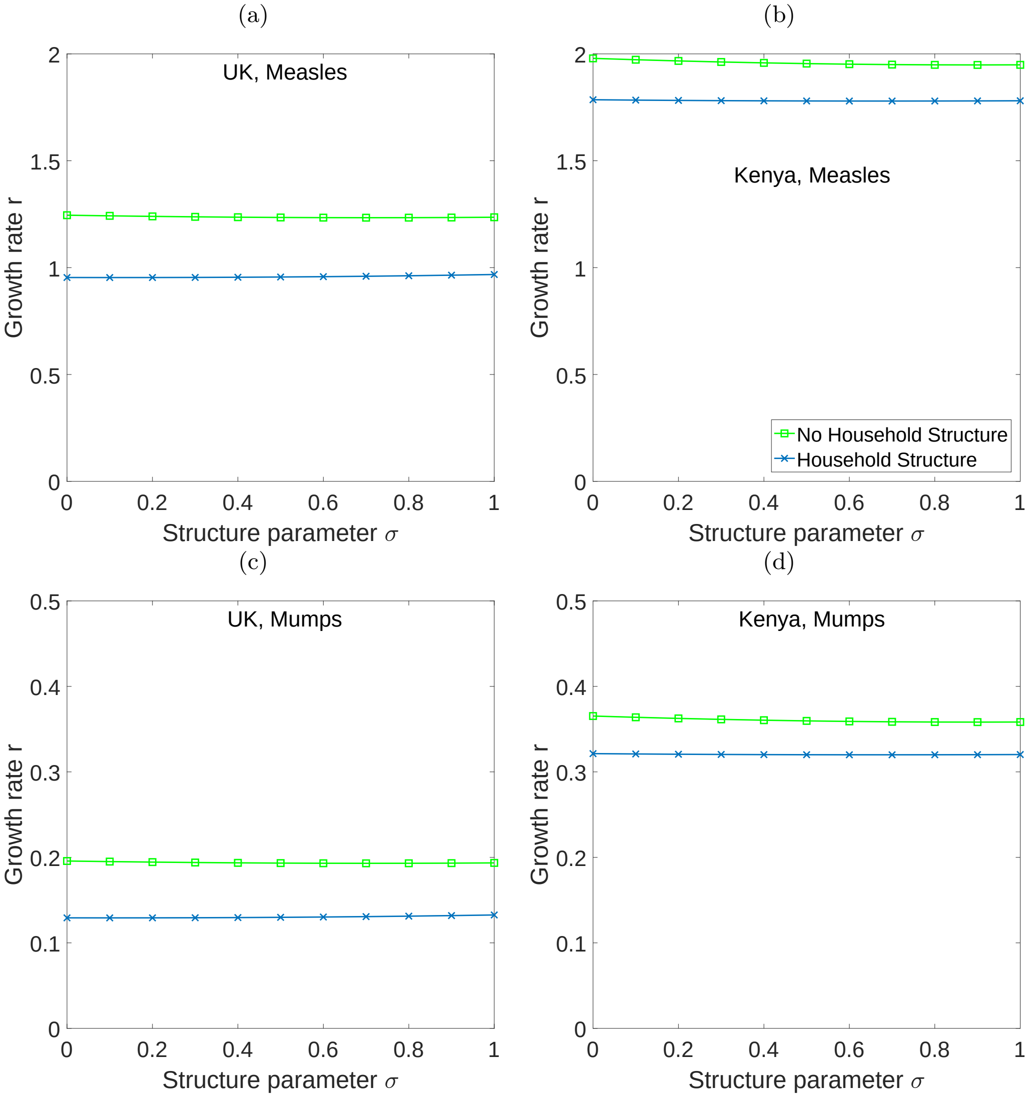

Figure 9: Early growth rate r as a function of structure parameter σ under UK- and Kenya-like demographic parameters for measles and mumps-like infectious parameters. Scaling the amount of age-structured compared to random transmission has limited impact on the early growth rate in all scenarios.

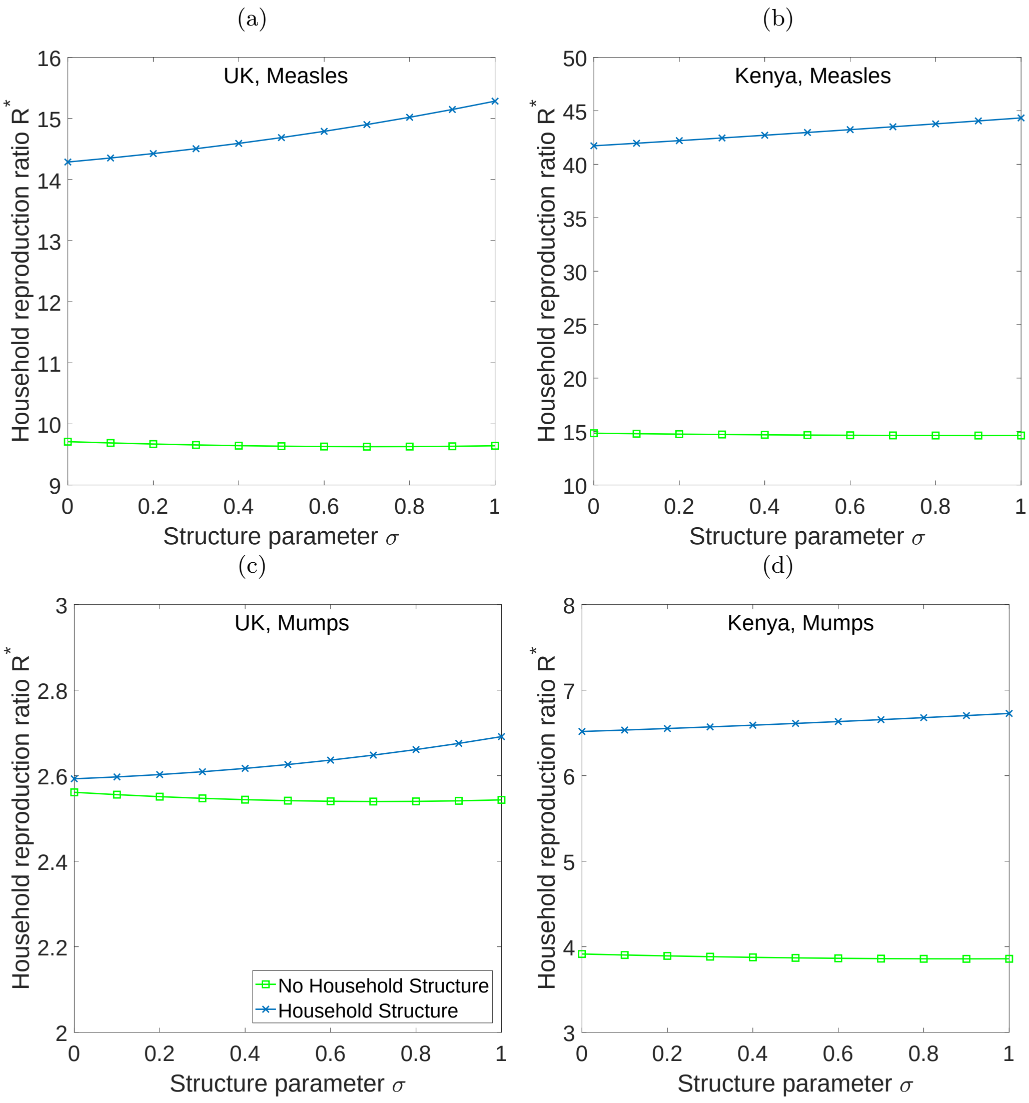

Figure 10: Household-level reproductive ratio R^* as a function of structure parameter σ under UK- and Kenya-like demographic parameters for measles and mumps-like infectious parameters.

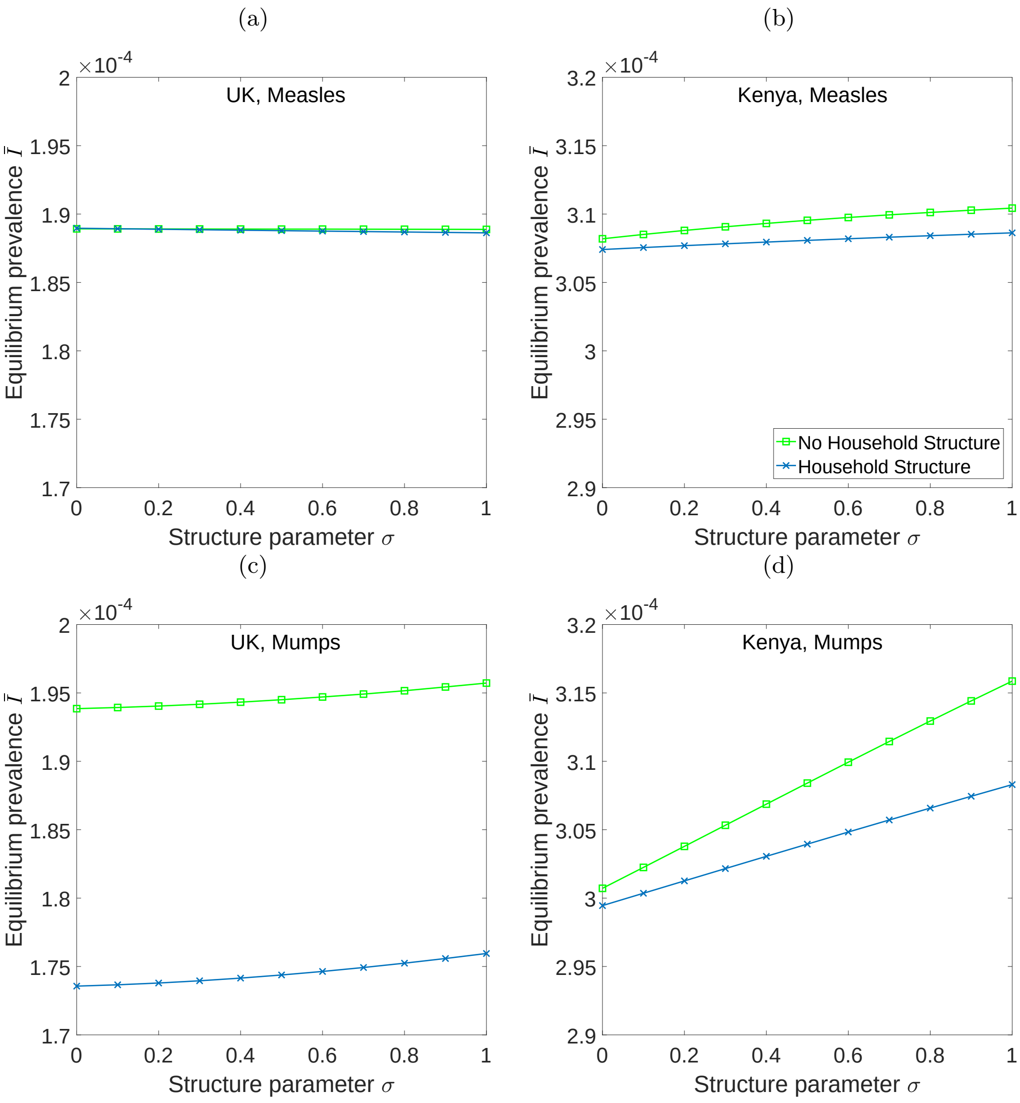

Figure 11: Equilibrium infectious prevalence $\bar{I}$ as a function of structure parameter σ under UK- and Kenya-like demographic parameters for measles and mumps-like infectious parameters.

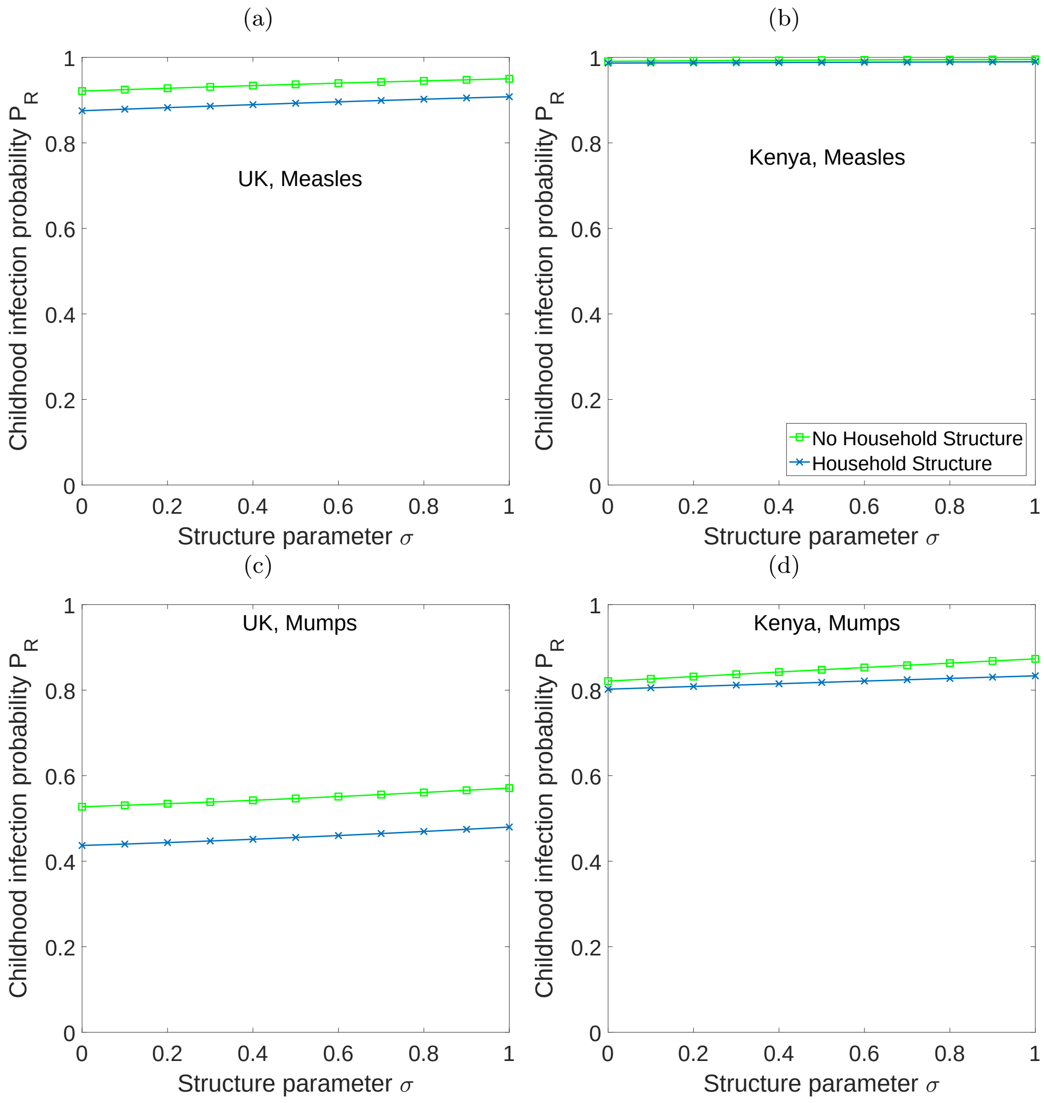

Figure 12: Endemic childhood infection probability P_R as a function of structure parameter σ under UK- and Kenya-like demographic parameters for measles and mumps-like infectious parameters.

4 Model Description

We now describe the two main components of the model: the epidemiological component and the demographic component, which to a large extent operate independently. Throughout the model description, the unit of time is one day.

4.1 Epidemiological model

The infectious process is modelled as a continuous-time Markov process with four events: recovery and infection via three routes. Recovery occurs at rate γI, where γ^{-1} is the expected length of the infectious period, and results in the transition $(S, I, R, k) \to (S, I, R+1, k)$. The three routes of infection all result in the same transmission event $(S, I, R, k) \to (S-1, I+1, R, k)$, but are associated with different rates. These routes and their associated rates are defined as follows:

1) The first route of infection is through internal contacts between members of the same household, who mix homogeneously with frequency dependent transmission. The choice of frequency-dependent transmission is motivated by the Bayesian analysis of household transmission data conducted by Cauchemez et al.[4], which suggests that within-household mixing is substantially closer to the frequency-dependent ideal than to the density-dependent ideal. Let d_{int} be the average time per day which individuals spend exposed to members of their own household, and let τ be the unit time rate of transmission of infection. Then the internal person-to-person transmission rate is given by $\beta_{\text{int}} = \tau d_{\text{int}}$ and, from the frequency dependence assumption, the internal transmission events occur at a rate $\beta_{\text{int}} SI/(N-1)$, where S, I and N refer to the susceptible individuals, infected individuals, and total occupancy of the household.

2) The second route of infection is through unstructured external contacts between members of distinct households. We assume that transmission occurs at the same unit time rate τ as for internal contacts, and define d_{ext} to be the average time per day which individuals spend exposed to individuals from outside their own household. Then unstructured transmission events occur at a rate $(1-\sigma)\beta_{\text{ext}} S\bar{I}$, where $\beta_{\text{ext}} = \tau d_{\text{ext}}$ and σ is a tuning parameter which defines the level of structure in the external contact process, and $\bar{I}$ is the mean level of infection in the population.

3) The third route of infection is through age-structured external contacts. We define K discrete age classes $C_1, ..., C_K$ such that every individual belongs to exactly one of these classes. Each individual in the household is exposed to infection based on an age-structured contact profile, and acts as a channel along which infection passes into the household. The age-structured contact profile for an individual in age class C_i is a row vector $\underline{D}_i$ of length K whose jth entry is the mean time per day in which an individual in age class C_i is exposed to members age class C_j. We stress that these entries record the total time spent in contact with members of C_j with individual contacts counted separately, so that the total exposure time per day may exceed one day if multiple simultaneous exposures are common. Taking these vectors together defines a $K \times K$ matrix of exposure durations $\boldsymbol{D}$ where $D_{i,j}$ is the average contact time per day between a single individual of age class C_i and the entire age class C_j. When multiplied by the transmission rate τ this defines a Who-Acquires-Infection-From-Whom (WAIFW) matrix, as is used in typical age-structured transmission models[8]. In our model the ages of the individuals in the household are not directly observable but are correlated with the demographic class (T); however, we can calculate the age-structured force of infection λ_T on individuals in a household in demographic class $T(N, k)$ by inferring a mean age profile for the household and applying the WAIFW-type dynamics to this profile. This calculation is somewhat involved and is covered in more depth in Section 6. Our model does not keep track of individual household members (in the sense that we do not know "who" is infectious) and so we assume that all members of the household act as channels of infection which can infect all of the others, which means the rate of infection in a household in state $(S, I, R, T(N, k))$ is $\lambda_T S$.

The household infectious disease model with demography assumes internal contacts to be homogeneous, so that only consider external contacts are considered in the construction of the contact profiles. We accordingly denote the resulting contact profile matrix $\boldsymbol{D}_{\text{ext}}$. Two of the special cases of the transmission model defined in Section 4 4.3 involve a profile which include both internal and external contacts, and we denote the

resulting contact profile matrix by $\boldsymbol{D}_{\mathrm{all}}$. The calculation of the contact profile matrices and the values used in our analysis are presented in Section 5.

4.2 Demographic model

The demographic model presents a simplified account of the evolution of a nuclear family, which we refer to as a household. The household initially consists of two individuals, the "parents". The parents reproduce, adding children to the household. At some point they stop reproducing, and after some amount of time the now-adult children start to leave the household in the same order that they were born. Once all the children are gone, the parents remain in the household for some period of time after which they are replaced by a new couple, and the process begins again. In this way we can maintain a fixed population size of households, but have a birth-rate that is higher than the death-rate due to the emigration of young-adults when they leave home if there isn't sufficient space in the population. Throughout we assume that couples who begin a household stay together for life and that there is zero mortality in childhood. For the purposes of our epidemic model, we also assume that new couples are drawn at randomly from the appropriate cross-section of the population, so that the couples starting new households are statistically identical to the children leaving home. With these assumptions in mind, we now give a formal description of the model.

Parameter	Description	UK-like population	Kenya-like population
T_B	Mean between-birth interval	1096 (=3 years)	1071 (=35.2 months)
k_B	Number of ticks between births	2	2
T_L	Mean age of child leaving home	9131 (=25 years)[5]	8291 (=22.7 years)
k_L	Number of ticks between last birth and first leaving	4	4
T_D	Life expectancy	29,220 (=80 years)	24,472 (=67 years)
k_R	Number of ticks between last child leaving and reset	5	5

Table 2: Parameter values for the demographic process; all times are given in days with appropriate conversions to a more human scale. The k values, which determine the Gamma distribution shape parameters, were chosen to produce reasonably . The between-birth interval for the UK was from the ONS[12] and for Kenya was from the DHS[9, 72]. The mean age at leaving home for the UK was from Eurostat[5] and for Kenya was from the DHS[9, 60]. The life expectancy for the UK was from the ONS[14] and for Kenya was from the KHDSS[16]. The parameter estimations are explained in more detail in Section 5.

The evolution of a household is defined in terms of four demographic phases and events which occur at the end of each phase, with the potential for some phases to occur multiple times in succession. The demographic class is defined uniquely by the pair (N, k), independent of the infectious state of the individuals within the household. The current phase is indexed by the value of k with specific values of k triggering the end-of-phase events which can cause a change in N or k. Because k increments at exponential time intervals, the demographic process is a continuous-time Markov chain, and the demographic events occur at Erlang distributed intervals. The parameters of the demographic process are listed in Table 2. We define the probability that a randomly chosen household produces N_C children over its lifetime to be $P_C[N_C]$, with expectation $\bar{N_C}$. We will always assume that there exists a maximum household size $N_{\max}$ such that $P_C[N-2] = 0$ for all $N = 2 + N_C \geq N_{\max}$. These distributions are listed for UK-like and Kenya-like populations in Table 3. This distribution for the number of children gives rise to a set of stopping probabilities $P_{\mathrm{stop}}[N] = P_C[N-2]/(1 - \sum_{n=0}^{N-3} P_C[n])$, defined as the probability that a household of total size N (including the parents) stops producing children. We note that $P_{\mathrm{stop}}[2] = P_C[0]$ and that $P_{\mathrm{stop}}[N] = 0$ for $N = 2 + N_C \geq N_{\max}$. The demographic events and associated k-phases are as follows:

1. Waiting for a child: $1 \leq k \leq k_B$. In this phase k increments at a rate k_B/T_B so that the expected length of the phase is T_B, the expected interval between births. When incrementing from $k = k_B$, a new (susceptible) child is added to the household which either stops producing children with probability

$P_{\text{stop}}[N]$ or repeats this phase (resetting $k = 1$) and has another child with probability $1 - P_{\text{stop}}[N]$. Thus at the end of this phase the status of the household either transitions $(S, I, R, k_B) \to (S+1, I, R, k_B + 1)$ with probability $P_{\text{stop}}[N]$ or transitions $(S, I, R, k_B) \to (S+1, I, R, 1)$ with probability $1 - P_{\text{stop}}[N]$.

	0	1	2	3	4(+)	5	6	7	8	9	10+
UK, ONS Data[13]	17	18	37	17	1						
Kenya, DHS[9]	1.9	3.8	9.8	12.9	15.0	13.2	11.9	10.8	7.8	5.0	7.9

Table 3: Percentage distribution of women by number of live-born children, which we use as a proxy for the distribution of children born to a pair of parents in our model. Data for the UK is from the ONS[13], data for Kenya is from the DHS[9, 71]. The parameter estimations are explained in more detail in Section 5.

2. Waiting for eldest child to mature: $k_B + 1 \leq k \leq k_B + k_L$. In this phase k increments at a rate $k_L/(T_L - (N-3)T_B)$ so that the expected length of the phase is $T_L - (N-3)T_B$. When exiting state $k = k_B + k_L$ the eldest child leaves the household. This child has $(N-3)$ younger siblings and thus has already lived through $(N-3)$ phases of expected length T_B, giving them an expected age of T_L when they leave home. If $N > 3$ the counter increments from $k_B + k_L$ to $k_B + k_L + 1$, entering the waiting period for the next child to leave home. If $N = 3$ the counter increments straight to $2k_B + k_L$, entering the waiting period for renewal of the household. Using the reasoning outlined in Section 2.5, the infectious status of the eldest child is chosen by considering the infectious status of the entire household and removing from consideration any adults who were recovered when the house was established at $T(2, 0) = 0$. We thus choose uniformly from the infectious status (S, I, R) of the household, discounting R and N by $2P_R$ when $R \geq 2$, P_R when $R = 1$, and 0 when $R = 0$. This procedure gives rise to a fairly complicated set of transition probabilities which are summarised in the following table:

Number of recovered individuals	Probability of transition		
	$(S, I, R) \to (S-1, I, R)$	$(S, I, R) \to (S, I-1, R)$	$(S, I, R) \to (S, I, R-1)$
$R = 0$	$\frac{S}{N}$	$\frac{I}{N}$	0
$R = 1$	$\frac{S}{N-P_R}$	$\frac{I}{N-P_R}$	$\frac{1-P_R}{N-P_R}$
$R \geq 2$	$\frac{S}{N-2P_R}$	$\frac{I}{N-2P_R}$	$\frac{R-2P_R}{N-2P_R}$

3. Waiting for other children to leave: $k_B + k_L + 1 \leq k \leq 2k_B + k_L$. In this phase k increments at a rate k_B/T_B so that the lengths of these intervals are distributed identically to those of the between-birth intervals. At the end of this interval the oldest child remaining in the household leaves home; the age of this child is distributed identically to the age of the first child to leave home. If $N > 3$ the counter transitions from $2k_B + k_L$ back to $k_B + k_L + 1$ and repeat this phase for the next oldest child, otherwise the counter increments to $2k_B + k_L + 1$, moving the household into the last phase of the demographics. The infectious status of the leaving child is chosen according to the same procedure as that of the eldest child to leave home.

4. Waiting for household replacement: $2k_B + k_L + 1 \leq k \leq 2k_B + k_L + k_R$. In this phase k increments at a rate k_R/T_R, where $T_R = T_D - (1 - P_C[0])T_L - \bar{N}_C T_B - T_L$ gives the expected time between a couple's eldest child leaving (if they have a child) and the couple's replacement by a new nuclear family. Note that this expectation includes couples who do not have any children and thus enter this phase immediately after leaving home. This formulation means that individuals are actively involved with epidemic processes for an average time that is equal to the life expectancy, T_D. When the old inhabitants are replaced the new couple are each drawn uniformly at random from the set of children leaving home (see Demographic Events 2 and 3). Leaving children are each either recovered (with probability P_R) or susceptible (with probability $1 - P_R$); the absence of infectious cases justified by the difference in timescales between the demographic process and the infectious period of the diseases of interested. Its calculation is detailed in Section 2.5. This new couple have children with probability

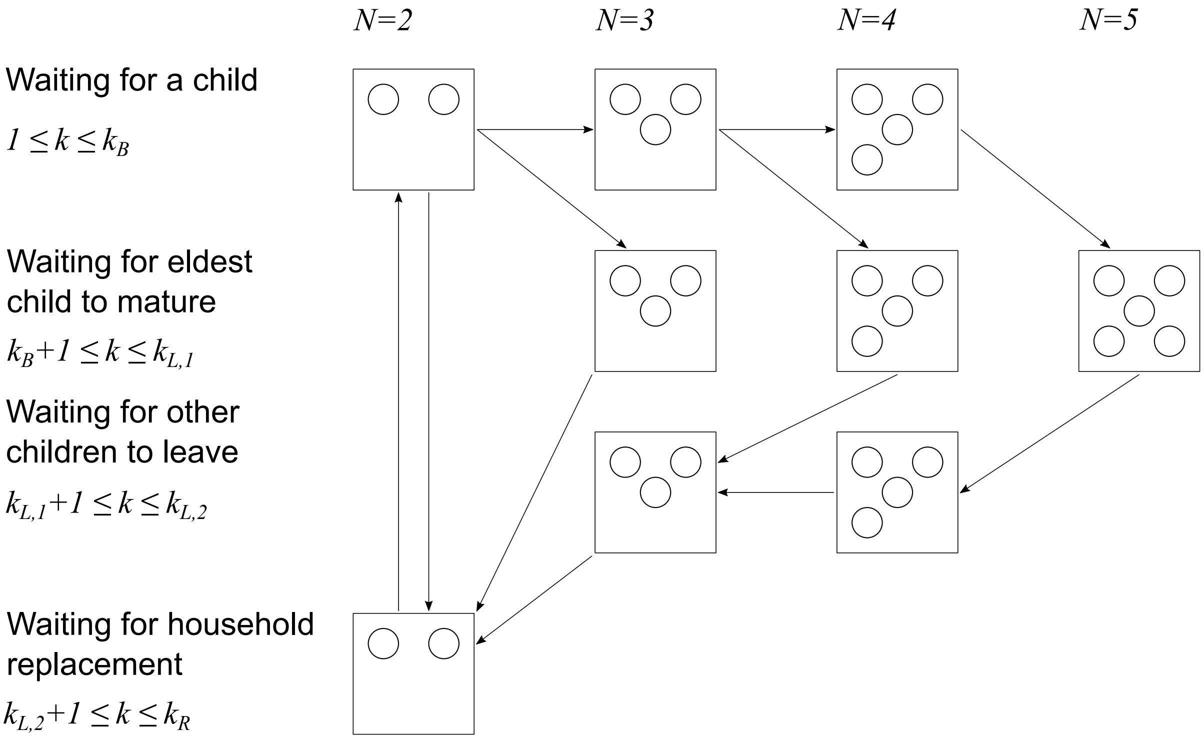

Figure 13: The state of the demographics is defined by the household size N and ticker state k. Each row corresponds to one phase of the household's lifespan, proceeding from birth phase to first leaving phase to second leaving phase to reset phase. The intervals between transitions are follow an Erlang distribution. Here the maximum household size is 5.

$1 - P_C[0]$, leading them into the first phase ($k = 1$), otherwise they move straight into the fourth phase ($k = 2k_B+k_L+1$). With these considerations in mind, we transition $(S, I, R, 2k_B+k_L+k_R) \to (s, 0, r, 1)$ with probability $P_C[0] \times (1-P_R)^s \times P_R^r \times (1+sr)$ or $(S, I, R, 2k_B+k_L+k_R) \to (s, 0, r, 2k_B+k_L+1)$ with probability $(1-P_C[0]) \times (1-P_R)^s \times P_R^r \times (1+sr)$.

The demographic model is illustrated schematically in Figure 13 for a system with maximum household size of $N = 5$ (or $N_C = 3$).

4.3 Special cases

The household infectious disease model with demography includes three standard transmission models that we consider as special cases. Setting $\sigma = 0$, $\beta_{\text{int}} = 0$, and $\beta_{\text{ext}} = \tau d_{\text{all}}$, where $d_{\text{all}} = d_{\text{int}} + d_{\text{ext}}$ is the total time per day which individuals spend exposed to other members of the population, defines a *random mixing model* equivalent to the standard homogeneous SIR model with frequency-dependent transmission, independent of age- and household-structure. Setting $\sigma = 1$ and $\beta_{\text{int}} = 0$ and using the contact profile $\boldsymbol{D}_{\text{all}}$ to define the transmission rates, defines a *purely age-structured model* in which transmission obeys WAIFW-type dynamics with no household structure. Finally, setting $\sigma = 0$ with $\beta_{\text{int}} = \tau d_{\text{int}}$ and $\beta_{\text{ext}} = \tau d_{\text{ext}}$ defines a *purely household-structured model* with two distinct levels of mixing but no consideration of age structure. All three cases retain the underlying demographic process outlined in Section 4 4.2, despite the differences in how population structure influences the transmission process. For ease of reference, we refer to the standard case with $\sigma = 1$ as the *full age- and household-structured model* or simply the full model. In the full model there is a distinction between internal and external mixing, with global mixing being entirely age-stratified.

5 Incorporating data sources

Modelling the events of the infectious process requires knowledge of the infection's per unit time transmission rate and its recovery rate, along with all the appropriate contact durations. The first two quantities are disease-specific, whereas the contact durations are features of the population. The recovery rate is the reciprocal of the mean infectious duration, which is directly measurable. Informed by the values reported by Anderson and May[1], we choose infectious periods of 7 days for measles and 8 days for mumps. To calculate the unit time transmission rate, we refer to Hope-Simpson[7], who provides empirical transmission probabilities for both measles and mumps amongst British children belonging to the same household. Denote the observed transmission probability by p and the mean per-day contact duration between children of the same household by d_C. Then the expected contact time between two children during the infectious period is $\gamma^{-1}d_C$ and so under the assumption of a Poisson transmission process

$$p = 1 - \exp(-\tau\gamma^{-1}d_C). \tag{12}$$

Rearranging Equation 12 gives

$$\tau = -\frac{\gamma}{d_C}\log(1-p). \tag{13}$$

This calculation requires an estimate of d_C, which can be obtained from contact survey data. In our results we used the value obtained from the POLYMOD study for the UK[11], taking the mean duration across all contacts with "Home" listed as a contact location in which both the study participant and their contact are aged 16 or under. We do not attempt to adjust for the five decades separating the populations of Hope-Simpson's study and the POLYMOD survey, although in a practical setting it is important that the contact duration and transmission probability be estimated from the same population.

It should be noted here that in the calculation of the transmission rates τ we have explicitly assumed that the infectious period of both diseases is a fixed interval (of duration γ^{-1}); for measles and mumps this is a reasonable assumption and hence provides an accurate approximation to the expected transmission rate across any given contact. However for modelling simplicity and to lower the dimensionality of the system we have a single infectious class with a fixed recovery rate, which leads to exponentially distributed infectious times. When we consider external and age-structured contacts, because we assume that these contacts occur at random, the exponentially distributed infectious times combined with the transmission rate τ still gives us the correct expected number of infection events. However, the repeated nature of contacts within a household together with the exponentially distributed infectious times leads to an under-estimation of the expected number of secondary cases. Given the potential for huge differences between households of the 1950's which underpins Hope-Simpson's observations[7], and the modern households that are the focus for this study, we feel this approximation is reasonable. Alternatively, given more up-to-date data on within-household transmission (and potentially about transmission to non-household members) we could use different transmission rates for the two settings. The option of using a more realistic Erlang distribution for the infectious period would lead to a large increase in the dimensionality of the system and would make many of the calculations infeasible.

The contact durations required for our model can be estimated from social contact survey data on the population of interest. Given a sample set of contact events with durations, age of the participants, and an indication of whether the participants share a household, we can calculate the following parameters by taking the average duration of contacts of the specified type:

1. d_{int}, the average time per day which individuals spend exposed to members of their own household.
2. d_{ext}, the average time per day which individuals spend exposed to individuals from outside their own household.
3. d_C, the average duration of contacts between two children within the same household.
4. $\boldsymbol{D}$, the contact profile matrix with D_{ij} telling us the average time per day which individuals in age class C_i spend exposed to individuals in age class C_j.

We estimate contact durations and profiles for the UK from the data obtained through the POLYMOD study[11], and for Kenya from that obtained through the Health and Demographic Surveillance System (HDSS) in Kilifi, coastal Kenya[10]. Both datasets are publicly available as supplementary material attached to their respective publications. The POLYMOD data does not state which of a participant's contacts share a household with them, but it does give a list of locations at which each contact was encountered. As an approximation, we will define any contact for which "Home" is listed as a location to be an internal contact, and all others to be external. The HDSS dataset specifies whether or not two contacts share a household so that we can directly classify internal and external contacts from the data. Ages are specified in the POLYMOD study to the nearest year, so that we can estimate a contact profile matrix using any set of age classes. We choose a relatively low number of age-classes to reduce the computational burden, and concentrate on younger age-groups given our focus of capturing the infection dynamics of childhood diseases. The HDSS study only asked participants to assign their contacts to one of six age classes: Infant (<1 year old), pre-school (1-5 years), primary school (6-15 years), secondary school (15-19 years), adult (20-49 years), and elderly (>50 years). The HDSS data can thus only be easily used in models with this set of age classes (or some aggregation of them), and so we use these for our Kenya-like population. The age classes used in our two models and their sizes as percentage distributions are listed in Table 4.

Population	C_1	C_2	C_3	C_4	C_5	C_6
UK-like	¡1	1-2	3-5	6-10	11-16	17-98
Percentage distribution	2%	2%	3%	9%	8%	77%
Kenya-like	¡1	1-5	6-15	16-19	20-50	>50
Percentage distribution	2%	10%	14%	2%	40%	31%

Table 4: Age classes used in our models of UK-like and Kenya-like populations along with their associated percentage distributions. Age class boundaries are in years. Percentages may not sum to one hundred due to rounding.

For a given set of contact survey results let M be the total number of study participants,and J be the total number of recorded contacts,with each contact reported by exactly one participant. The data associated with the jth recorded contact can be written as the vector $(m_j, d_j, L_j, a_j^1, a_j^2)$, where $m_j \in 1, ..., M$ encodes the identity of the study participant who reported the contact, d_j is the duration of the contact event, L_j encodes the location of the contact, a_j^1 is the age of the study participant, and a_j^2 is the age of their contact. Specifically, we will use the encoding $L = 0$ for internal contacts and $L = 1$ for external contacts. Then the mean total exposure time experienced by individuals of age class C_k to individuals of age class C_l in location L is given by

$$\frac{1}{M} \sum_{m=1}^{M} \sum_{j=1}^{J} d_j \mathbf{1}_{m_j=m} \mathbf{1}_{L_j=L} \mathbf{1}_{a_j^1 \in C_k} \mathbf{1}_{a_j^2 \in C_l}, \tag{14}$$

where $\mathbf{1}$ is the indicator function equal to 1 if the given condition is satisfied and zero otherwise. Using this formula, we obtain the equations

$$d_{\text{int}} = \frac{1}{M} \sum_{m=1}^{M} \sum_{j=1}^{J} d_j \mathbf{1}_{m_j=m} \mathbf{1}_{L_j=0} \tag{15}$$

$$d_{\text{ext}} = \frac{1}{M} \sum_{m=1}^{M} \sum_{j=1}^{J} d_j \mathbf{1}_{m_j=m} \mathbf{1}_{L_j=1} \tag{16}$$

$$D_{ij} = \frac{1}{M} \sum_{m=1}^{M} \sum_{j=1}^{J} d_j \mathbf{1}_{m_j=m} \mathbf{1}_{L_j=1} \mathbf{1}_{a_j^1 \in C_k} \mathbf{1}_{a_j^2 \in C_l}. \tag{17}$$

In the purely age-structured model, the age-structured contact profiles are required across the entire population rather than just across the external contacts. The (i, j)th entry of the contact profile matrix is then

given by

$$\frac{1}{M}\sum_{m=1}^{M}\sum_{j=1}^{J} d_j \mathbf{1}_{m_j=m}\mathbf{1}_{a_j^1\in C_k}\mathbf{1}_{a_j^2\in C_l}, \tag{18}$$

where we have removed the location-based restriction.

The average child-to-child contact duration d_C is the mean duration of all internal (household) contacts in which both the study participant and their contact belong to one of the "child" age classes (C_1 to C_5 of the UK-like population and C_1 to C_4 of the Kenya-like population). We will denote the union of the "child" age classes by $\tilde{C}$. Note that this corresponds to the mean length of a single contact, rather than of a total exposure time across multiple contacts, so the average is taken across all contact events rather than across all study participants. This gives rise to the formula

$$d_c = \frac{\sum_{j=1}^{J} d_j \mathbf{1}_{L_j=0}\mathbf{1}_{a_j^1\in\tilde{C}}\mathbf{1}_{a_j^2\in\tilde{C}}}{\sum_{j=1}^{J} \mathbf{1}_{L_j=0}\mathbf{1}_{a_j^1\in\tilde{C}}\mathbf{1}_{a_j^2\in\tilde{C}}}. \tag{19}$$

The values of the daily exposure durations d_{int}, d_{ext}, and d_{all} are listed in Table 5. The age class-stratified exposure matrices are stated in Tables 7 and 8 and visualised in Figure 14. In the UK-like population, younger children tend to experience low levels of interaction with anyone outside the "adult" age class C_6, with the individuals they do interact with presumably being parents and other carers. Assortative mixing only becomes a substantial factor once children enter age class C_4, covering ages 6-10. Interactions in the Kenya-like population are less assortative than in the UK but individuals spend longer exposed to other members of the population, particularly those from outside the household. Table 6 lists class-stratified exposure durations under the assumption of homogeneous mixing, calculated by multiplying the relevant location-stratified exposure duration by the proportion of the population in the specified age class. Comparing the resulting profiles by the matrices in Tables 7 and 8 makes it clear whether age-structured mixing increases or decreases the level of contact between a given pair of age classes.

Exposure parameter	UK-like population	Kenya-like population
d_{int}	0.382	0.445
d_{ext}	0.397	0.746
d_{all}	0.779	1.191

Table 5: Daily exposure durations by location. UK values are derived from the POLYMOD study[11], Kenya values are derived from Kiti et. al.[10].

	C_1	C_2	C_3	C_4	C_5	C_6
UK, external contacts	0.007	0.007	0.013	0.037	0.031	0.303
UK, all contacts	0.013	0.013	0.026	0.072	0.060	0.594
Kenya, external contacts	0.016	0.078	0.103	0.018	0.299	0.231
Kenya, all contacts	0.025	0.125	0.165	0.029	0.478	0.369

Table 6: Expected daily exposure times to each class under homogeneous mixing, obtained by multiplying the distributions in Table 4 by the exposure durations in Table 5. For each population-location combination, the exposure profile is the same for each age class.

	$\boldsymbol{D}_{\text{ext}}$					
	C_1	C_2	C_3	C_4	C_5	C_6
C_1	0.030	0.000	0.000	0.007	0.006	0.072
C_2	0.016	0.031	0.042	0.019	0.000	0.125
C_3	0.000	0.013	0.089	0.023	0.001	0.099
C_4	0.000	0.002	0.018	0.501	0.020	0.150
C_5	0.000	0.001	0.002	0.039	0.573	0.145
C_6	0.001	0.001	0.004	0.022	0.013	0.302

	$\boldsymbol{D}_{\text{all}}$					
	C_1	C_2	C_3	C_4	C_5	C_6
C_1	0.031	0.009	0.027	0.138	0.060	0.508
C_2	0.026	0.042	0.073	0.097	0.033	0.410
C_3	0.011	0.035	0.111	0.102	0.026	0.416
C_4	0.003	0.007	0.052	0.601	0.099	0.481
C_5	0.002	0.008	0.013	0.104	0.696	0.411
C_6	0.003	0.007	0.019	0.067	0.061	0.525

Table 7: UK age-stratified matrices $\boldsymbol{D}_{\text{ext}}$ and $\boldsymbol{D}_{\text{all}}$ of daily exposure durations across external and all contacts respectively. These are derived from the data provided by the study POLYMOD study in the UK [11]. The ith row is the average daily contact profile for a member of age class C_i. This matrix is visualised in Figure 14a and 14c

	$\boldsymbol{D}_{\text{ext}}$					
	C_1	C_2	C_3	C_4	C_5	C_6
C_1	0.009	0.079	0.157	0.049	0.128	0.033
C_2	0.018	0.190	0.199	0.054	0.136	0.040
C_3	0.022	0.143	0.394	0.108	0.122	0.032
C_4	0.022	0.082	0.240	0.272	0.222	0.041
C_5	0.027	0.083	0.114	0.095	0.412	0.120
C_6	0.015	0.051	0.097	0.057	0.291	0.111

	$\boldsymbol{D}_{\text{all}}$					
	C_1	C_2	C_3	C_4	C_5	C_6
C_1	0.015	0.219	0.354	0.099	0.317	0.069
C_2	0.042	0.307	0.455	0.109	0.309	0.082
C_3	0.040	0.276	0.610	0.152	0.275	0.065
C_4	0.034	0.143	0.376	0.322	0.341	0.085
C_5	0.048	0.190	0.232	0.142	0.524	0.140
C_6	0.028	0.113	0.176	0.102	0.390	0.131

Table 8: Kenyan age-stratified matrices $\boldsymbol{D}_{\text{ext}}$ and $\boldsymbol{D}_{\text{all}}$ of daily exposure durations across external and all contacts respectively. These are derived from the data provided by the study of Kiti et. al. [10] in coastal Kenya. The ith row is the average daily contact profile for a member of age class C_i. This matrix is visualised in Figure 14b.

The demographic parameters can be fitted using data available from statistical surveys. Fitting the Erlang shape parameters k_B, k_L, and k_R would require detailed knowledge of the waiting time distributions, and so we choose values which reduce the variance of these times relative to an exponential distribution whilst still keeping the total number of states in the system relatively low. The choices of expectation parameters T_B, T_L, and T_D are informed by data.

For the UK, we estimate T_B as approximately 3 years based on the estimates of median interval between births provided by the Office for National Statistics (ONS)[12]. In 2017 these were 36 months between first and second birth, 35 between second and third, and 34 between third and fourth. In the absence of more information, we assume that the median gives a good approximation of the mean value. The average age at leaving the parental household T_L is provided directly by Eurostat[5]. The choice of 80 years for the life expectancy for T_D is motivated by the life tables for England provided by the ONS, in which children born in England in 2016 are predicted to survive 79.46 (male) and 83.10 (female) years. Given our focus on childhood infections, we believe that the life expectancy within the model should have relatively limited impact.

For Kenya, our estimates of T_B and T_L are from the Kenya Demographic and Health Survey (DHS) 2014[9]. The median birth interval is estimated to be 35.2 months in the coastal region where Kilifi is located[9, 72], and as with the UK we will assume this gives a good approximation of the mean value. We approximate average age at leaving home by the average age at first marriage. The DHS provides the median age at first marriage stratified by county, and for Kilifi provides values of 18.9 for women and 24.8 for men[9, 60]. Taking an unweighted average and assuming that the mean is well approximated by the median, we use 21.9 as our average age at leaving. The profile of the KHDSS by Scott et al. lists life expectancies of 69.5 for men and 75.4 for women[16], and so we set T_D to be the mean of these two values, 72.45.

The distribution P_C of the total number of children born in a household over its lifetime can be estimated

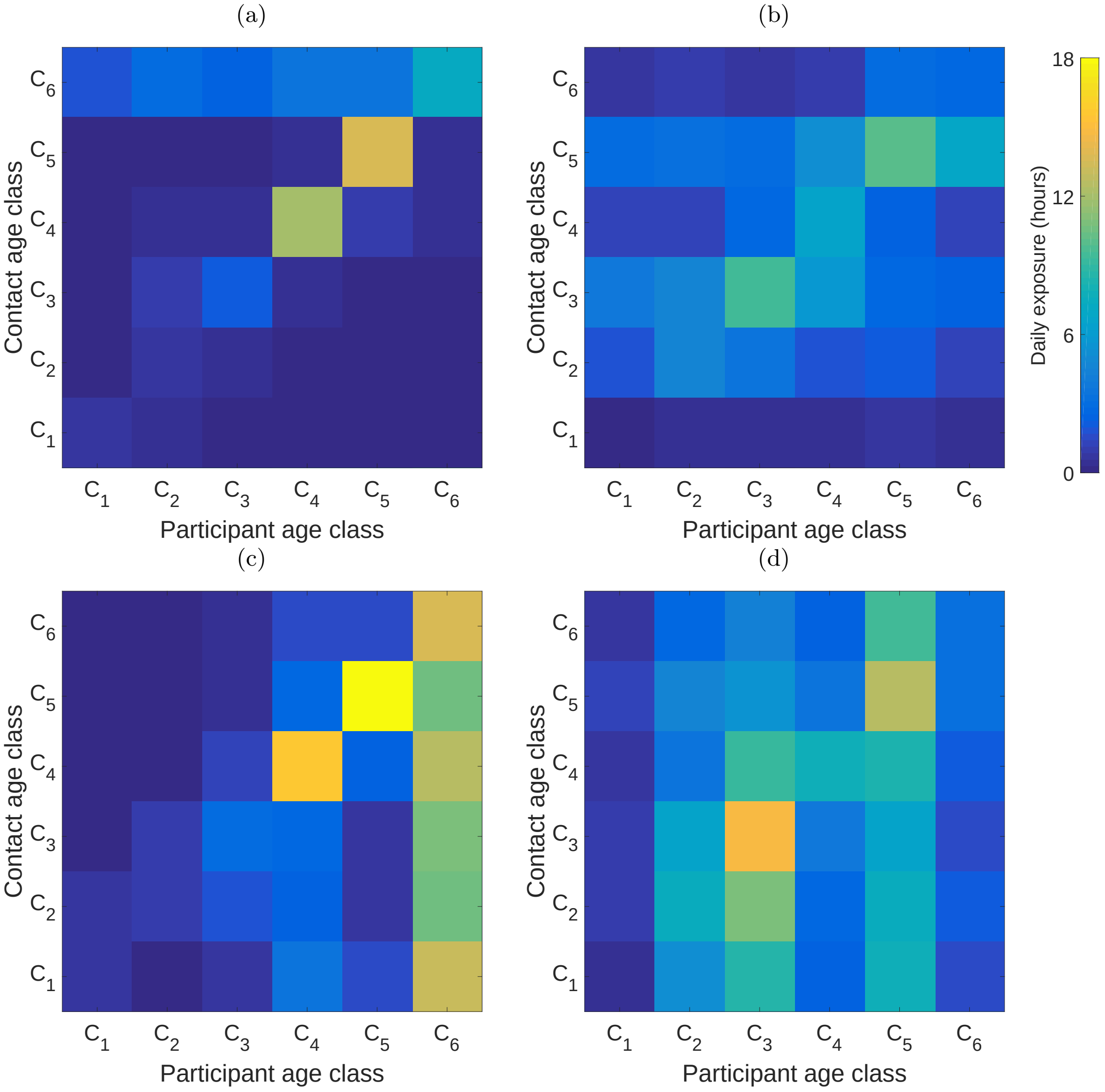

Figure 14: Average exposure duration matrices based on: (graph a) external contacts in the UK, ignoring contacts made within the household; (graph b) external contacts in Kenya, ignoring household contacts; (graph c) all contacts in the UK, including both household and external contacts; and (graph d) all contacts in Kenya. Profiles are derived from the data of the POLYMOD study for the UK [11] and the study of Kiti et. al. for Kenya[10].

from age-stratified records of the number of children born to women in the population of interest. The ONS provides a percentage distribution of the number of children born to women who were born in 1970[13]. Assuming that this cohort has now stopped having children, we can take this distribution to be the total number of children born to a woman over a lifetime, which we use as P_C in our UK-like population. For the Kenya-like population, we use the percentage distribution of number of children born to women aged 44 to 49 years old, which is provided by the DHS[9, 71]. These distributions are listed in Table 3. The data sources list the maximum number of children as 4+ and 10+ respectively, which in our model we replace with their exact values. Based on the values we assign to T_B, T_L, and P_C in the Kenya-like population, we see that the maximum number of children in a household is 10 but the age at leaving home for an elder sibling T_L is less than $8T_B$, meaning that for families with nine or ten children the time intervals in the second phase of the demographic model will have negative length. To avoid this problem we aggregate the entries of P_C corresponding to $N_C > \frac{T_L}{T_B} + 1$ so that the age of the eldest child when the youngest child is born, $(N_C - 1)T_B$, is less than the expected age at leaving T_L. In the case of Kenya this means we set the maximum number of children to be 8, with probability equal to the DHS-derived probability that 8 or more children are born.

6 Calculation of the age-structured rate of infection $\underline{F}$

In this section we explain in detail the calculation of the age-structured rate of infection, denoted $\underline{\lambda}$. This is a vector indexed by the demographic class $T(N,k)$. The Tth entry λ_T is the age-structured force of infection experienced by and individual residing within a household in demographic class T.

The contact profile matrix $\boldsymbol{D}$ and transmission rate τ define mixing between age classes, so that the rate at which an infectious individual of age class C_j transmits infection to an individual of age class C_i is τD_{ij}. We define age-structured mixing on the level of households by assuming that each member of the household forms an age-structured channel along which infection can leave and enter the household. Because our model does not directly keep track of the ages and infection status of each individual household member, we assume that these channels result in exposure to the total amount of infection within the household. Let $E_{T,i}$ be the expected number of individuals in age class C_i belonging to a household in demographic class T. The calculation of this expectation is outlined in Section 6.1. The expected total time which the members of a household in demographic class T are exposed to individuals of age class C_j is then

$$\sum_i E_{T,i} D_{i,j}. \tag{20}$$

Given the probability H_T that a household is in demographic class T, we can define

$$\tilde{E}_{i,T} = \frac{H_T E_{T,i}}{\sum\limits_T H_T E_{T,i}}, \tag{21}$$

the probability that an individual of age class C_i belongs to a household in demographic class T. The expected amount of infection in an age class C_j individual's household is thus

$$\sum_U \tilde{E}_{j,U} \bar{I}_U. \tag{22}$$

This quantity can be interpreted as the household-level prevalence associated with age class C_j and is plotted in figs. 3 and 4. Taking Equations 20 and 22 together, the expected per-member rate of transmission to a household in demographic class T is given by

$$\lambda_T = \sum_{i,j} \frac{E_{T,i}}{N_T} \beta^{\text{ext}}_{i,j} \sum_U H_U E_{U,j} \frac{\bar{I}_U}{P_j}. \tag{23}$$

Using $\underline{I}$ to denote the vector with Tth entry $\bar{I}_T$, Equation 23 can be rewritten as the matrix expression

$$\underline{\lambda} = \tau \boldsymbol{E}\boldsymbol{D}\tilde{\boldsymbol{E}}\underline{I}. \tag{24}$$

6.1 Calculation of $E_{T,i}$

In the following calculation we will take advantage of the fact that the two age partitions we defined in Table 4 include a broadly defined adult range (16-98 years in the UK-like population), with the Kenya-like population further dividing this into adults of roughly child-rearing age (20-50 years) and elders (> 50 years). We make the approximation that in the UK-like population the "parents" of the household always belong to the adult class, and that in the Kenya-like population they belong to the adult class for states with $k \leq 2k_B + k_L$ and to the elder class for states with $k > 2k_B + k_L$, so that the transition occurs when the last of their children leave home. Our calculation can thus be restricted to the expected number of children of age class C_i in a household in demographic class T, which we denote $\tilde{E}_{T,i}$. For the UK-like population we then have $E_{T,6} = \tilde{E}_{T,6} + 2$ for all T, whereas for the Kenya-like population we have $E_{T,5} = \tilde{E}_{T,5} + 2$ for all T with $k(T) \leq 2k_B + k_L$, and $E_{T,6} = \tilde{E}_{T,6} + 2$ for all T with $k(T) > 2k_B + k_L$, with $E_{T,i} = \tilde{E}_{T,i}$ elsewhere in both cases.

In the calculations that follow we define the instantaneous age profile $x = (x_1, ..., x_{N_C})$ to be the exact ages of the $N_C = N(T) - 2$ children in a household, taking the oldest first (i.e. $x_1 > x_2 > \ldots > x_{N_C}$). Our approach is then as follows: derive an expression for the distribution of $(x_1, ..., x_{N_C})$, and subsequently integrate this expression to get the expected number of children in each age class.

Distribution of the instantaneous age profile. Although the elements of the instantaneous age profile x are heavily correlated, the time intervals between births are independently Erlang distributed. Define $y = (y_1, ..., y_{N_C})$ with $y_i = x_i - x_{i+1}$ for $i = 1, ..., N_C - 1$ and $y_{N_C} = x_{N_C}$. This vector is uniquely determined by x. The first $N_C - 1$ elements of y are realisations of the inter-birth waiting time from the demographic model, making them independently Erlang distributed with shape parameter k_B and rate parameter k_B/T_B. The final element y_{N_C} is the age of the youngest child, whose distribution is dependent on the value of k as follows:

1. $1 \leq k \leq k_B$. In this case the youngest child's life covers the first k ticks of the first phase of the demographic model and so y_{N_C} is Erlang distributed with shape parameter $k - 1$ and rate parameter k_B/T_B.

2. $k_B + 1 \leq k \leq k_B + k_L$. In this case the youngest child's life covers the first k ticks of the second phase of the demographic model and so y_{N_C} is Erlang distributed with shape parameter $k - k_B - 1$ and rate parameter $k_L/(T_L - (N-3)T_N)$.

3. $k_B + k_L + 1 \leq k \leq 2k_B + k_L$. This case is more complicated because the demographic class $T(N, k)$ does not tell us how many elder siblings the child has had and thus how many times this phase of the demographics has already been repeated. If M elder siblings have already left the household, the youngest child will have experienced k_L time intervals at rate $k_L/(T_L - (N-3)T_B)$ and $(M-1) * k_L + k - 1$ at rate k_B/T_B, meaning that its age will be hypoexponentially distributed with a parameter vector consisting of k_L copies of the first rate and $(M-1) * k_L + k - 1$ copies of the second. Denote this parameter vector by $\underline{\Lambda}_M$.Then the probability of the youngest child being of age y_{N_C} is

$$\sum_{M=1}^{N_{\max}-N} P_{\text{stop}}[M + N] f_{\text{Hypoexp}}(y_{N_C} | \underline{\Lambda}_M), \tag{25}$$

   where $f_{\text{Hypoexp}}(\cdot | \underline{\Lambda}_M)$ is the probability density function of the hypoexponential distribution with parameter vector $\underline{\Lambda}_M$.

Since there are no children present in the fourth phase of the demographics, these three cases cover all possibilities.

The probability density function of x for a household in demographic class $T(N, k)$ is thus

$$f_{\text{Age}}(\underline{x}|T) = \begin{cases} f_{\text{Erl}}(x_{N_C} | k, \frac{k_B}{T_B}) \prod_{i=1}^{N_C - 1} f_{\text{Erl}}(x_i - x_{i+1} | k_B, \frac{k_B}{T_B}) & 1 \leq k \leq k_B \\ f_{\text{Erl}}(x_{N_C} | k - k_B - 1, \dfrac{k_L}{T_L - (N-3)T_B}) \prod_{i=1}^{N_C - 1} f_{\text{Erl}}(x_i - x_{i+1} | k_B, \frac{k_B}{T_B}) & k_B + 1 \leq k \leq k_B + k_L \\ \sum_{M=1}^{N_{\max}-N} P_{\text{stop}}[M + N] f_{\text{Hypoexp}}(y_{N_C} | \underline{\Lambda}) \prod_{i=1}^{N_C - 1} f_{\text{Erl}}(x_i - x_{i+1} | k_B, \frac{k_B}{T_B}) & k_B + k_{L,1} + 1 \leq k \leq 2k_B + k_L. \end{cases} \tag{26}$$

which relies on both Erlang (f_{Erl}) and hypoexponential (f_{Hypoexp}) probability density functions. Because the Erlang and hypoexponential distributions are only defined for positive waiting times, the distribution f_{Age} is only defined when $x_1 < x_2 < ... < x_{N_C}$, and so we set $f_{\text{Age}}(\underline{x}|T) = 0$ whenever this is not the case. For ease of notation we use $f_{\text{Age}}(x_1, ..., x_{N_C})$ to denote the probability density evaluated at the vector $(x_1, ..., x_{N_C})$, dropping the internal set of brackets. Notice that for all $n < N_C$,

$$f_{\text{Age}}(x_1, \ldots x_n, n_{n+1}, \ldots, x_{N_C}) = f_{\text{Age}}(x_{n+1}, \ldots, x_{N_C}) \prod_{i=1}^{n} f_{\text{Erl}}(x_i - x_{i+1} | k_B, \frac{k_B}{T_B}), \tag{27}$$

a fact which we will use in the calculation of $\tilde{E}_{T,i}$.

Age class probabilities. Let L_i and U_i be respectively the lower and upper boundaries of the age class C_i. Then $E_{T,i}$ is precisely the expected number of children between ages L_i and U_i within a household in demographic class T. This is the sum over the N_C children of the probability that the nth child is in the age interval (L_i, U_i), giving rise to the formula

$$\tilde{E}_{T,i} = \sum_{n=1}^{N_C} \int_{\mathbb{R}^{N_C}} f_{\mathrm{Age}}(\underline{x}|T) \mathbf{1}_{L_i < x_n < U_i} \mathrm{d}\underline{x}, \tag{28}$$

with $\mathbf{1}$ being the indicator function. Define $E_n(a, b)$ to be nth term of this sum,

$$E_n(L_i, U_i) = \int_{\mathbb{R}^N} f_{\mathrm{Age}}(x|T) \mathbf{1}_{L_i < x_n < U_i} \, \mathrm{d}x \tag{29}$$

$$= \int_0^\infty ... \int_{\min(L_i, x_{n+1})}^{U_i} ... \int_{x_2}^\infty f_{\mathrm{Age}}(\underline{x}|T) \, \mathrm{d}x_1 ... \mathrm{d}x_n ... \mathrm{d}x_{N_C}, \tag{30}$$

with the lower limits of each integral arising from the fact that f_{Age} is zero unless $x_1 > x_2 > ... > x_{N_C}$. Because the age of the nth child is independent of that of its elder siblings, the interior $n-1$ integrals evaluate to 1 and we obtain

$$E_n(L_i, U_i) = \int_0^\infty ... \int_{\min(a, x_{n+1})}^{b} f_{\mathrm{Age}}(x_n, ..., x_{N_C}|T) \mathrm{d}x_n ... \mathrm{d}x_{N_C}, \tag{31}$$

which we compute by Monte Carlo integration. Although this calculation of $\tilde{E}_{T,i}$ is computationally intensive, we note that it only needs to be performed once for any given population parameters.

References

[1] Roy M. Anderson and Robert M. May. *Infectious Diseases of Humans: Dynamics and Control*. Oxford University Press, 1992.

[2] Frank Ball, Denis Mollison, and Gianpaolo Scalia-Tomba. Epidemics with two levels of mixing. *Ann. Appl. Probab.*, 7(1):46–89, 02 1997.

[3] Cross Paul C., Lloyd-Smith James O., Johnson Philip L. F., and Getz Wayne M. Duelling timescales of host movement and disease recovery determine invasion of disease in structured populations. *Ecology Letters*, 8(6):587–595.

[4] Simon Cauchemez, F Carrat, C Viboud, AJ Valleron, and PY Boelle. A bayesian mcmc approach to study transmission of influenza: application to household longitudinal data. *Statistics in medicine*, 23(22):3469–3487, 2004.

[5] Eurostat, the statistical office of the European Union. Estimated average age of young people leaving the parental household by sex. `http://appsso.eurostat.ec.europa.eu/nui/show.do?dataset=yth_demo_030`, 2017.

[6] Geoffrey R. Grimmet and David R. Stirzaker. *Probability and Random Processes*. Oxford University Press, third edition, 2001.

[7] R.E. Hope-Simpson. Infectiousness of communicable diseases in the household: (measles, chickenpox and mumps). *The Lancet*, 260(6734):549 – 554, 1952. Originally published as Volume 2, Issue 6734.

[8] M. J. Keeling and P. Rohani. *Modeling Infectious Diseases in Humans and Animals*. Princeton University Press, 2007.

[9] Kenya National Bureau of Statistics, Ministry of Health/Kenya, National AIDS Control Council/Kenya, Kenya Medical Research Institute, and National Council for Population and Development/Kenya. Kenya demographic and health survey 2014. `http://dhsprogram.com/pubs/pdf/FR308/FR308.pdf`, 2015.

[10] Moses Chapa Kiti, Timothy Muiruri Kinyanjui, Dorothy Chelagat Koech, Patrick Kiio Munywoki, Graham Francis Medley, and David James Nokes. Quantifying age-related rates of social contact using diaries in a rural coastal population of kenya. *PLOS ONE*, 9(8):1–9, 08 2014.

[11] Joël Mossong, Niel Hens, Mark Jit, Philippe Beutels, Kari Auranen, Rafael Mikolajczyk, Marco Massari, Stefania Salmaso, Gianpaolo Scalia Tomba, Jacco Wallinga, Janneke Heijne, Malgorzata Sadkowska-Todys, Magdalena Rosinska, and W. John Edmunds. Social contacts and mixing patterns relevant to the spread of infectious diseases. *PLOS Medicine*, 5(3):1–1, 03 2008.

[12] Office for National Statistics. Births by parents' characteristics. `https://www.ons.gov.uk/peoplepopulationandcommunity/birthsdeathsandmarriages/livebirths/datasets/birthsbyparentscharacteristics`, 2017.

[13] Office for National Statistics. Childbearing for women born in different years. `https://www.ons.gov.uk/peoplepopulationandcommunity/birthsdeathsandmarriages/conceptionandfertilityrates/datasets/childbearingforwomenbornindifferentyearsreferencetable`, 2017.

[14] Office for National Statistics. National life tables: England. `https://www.ons.gov.uk/peoplepopulationandcommunity/birthsdeathsandmarriages/lifeexpectancies/datasets/nationallifetablesenglandreferencetables`, 2017.

[15] Joshua V. Ross, Thomas House, and Matt J. Keeling. Calculation of disease dynamics in a population of households. *PLOS ONE*, 5(3):1–9, 03 2010.

[16] J Anthony G Scott, Evasius Bauni, Jennifer C Moisi, John Ojal, Hellen Gatakaa, Christopher Nyundo, Catherine S Molyneux, Francis Kombe, Benjamin Tsofa, Kevin Marsh, Norbert Peshu, and Thomas N Williams. Profile: The kilifi health and demographic surveillance system (khdss). *International Journal of Epidemiology*, 41(3):650–657, 2012.
